# Enhancing carbon dioxide gas-diffusion electrolysis by creating a hydrophobic catalyst microenvironment

**DOI:** 10.1038/s41467-020-20397-5

**Published:** 2021-01-08

**Authors:** Zhuo Xing, Lin Hu, Donald S. Ripatti, Xun Hu, Xiaofeng Feng

**Affiliations:** 1 School of Material Science and Engineering, University of Jinan, Jinan, China; 2 Department of Physics, University of Central Florida, Orlando, FL USA; 3 Department of Materials Science and Engineering, University of Central Florida, Orlando, FL USA; 4 Polymers Science and Material Chemistry, Exponent Inc, Menlo Park, CA USA; 5 Renewable Energy and Chemical Transformations (REACT) Cluster, University of Central Florida, Orlando, FL USA

**Keywords:** Catalytic mechanisms, Electrocatalysis, Renewable energy

## Abstract

Electroreduction of carbon dioxide (CO_2_) over copper-based catalysts provides an attractive approach for sustainable fuel production. While efforts are focused on developing catalytic materials, it is also critical to understand and control the microenvironment around catalytic sites, which can mediate the transport of reaction species and influence reaction pathways. Here, we show that a hydrophobic microenvironment can significantly enhance CO_2_ gas-diffusion electrolysis. For proof-of-concept, we use commercial copper nanoparticles and disperse hydrophobic polytetrafluoroethylene (PTFE) nanoparticles inside the catalyst layer. Consequently, the PTFE-added electrode achieves a greatly improved activity and Faradaic efficiency for CO_2_ reduction, with a partial current density >250 mA cm^−2^ and a single-pass conversion of 14% at moderate potentials, which are around twice that of a regular electrode without added PTFE. The improvement is attributed to a balanced gas/liquid microenvironment that reduces the diffusion layer thickness, accelerates CO_2_ mass transport, and increases CO_2_ local concentration for the electrolysis.

## Abstract (web summary)

The local environment for carbon dioxide electrochemical reduction is a key element to improve the efficiency of catalytic sites. Here, the authors demonstrate substantial gain of the catalyst electrochemical activity through the adoption of a hydrophobic catalyst microenvironment.

## Introduction

Because of the limited reserves of fossil fuels, there is a rising demand for renewable energy technologies that can reduce our dependence on fossil fuels and address the anthropogenic climate change^1^. A promising approach is to power the synthesis of fuels and chemicals from naturally abundant resources using renewable electricity^2^. Such electrosynthesis processes are compatible with the intermittent supply of electricity from renewable resources, such as solar or wind, and can enable sustainable production of fuels and chemicals^3^. Accordingly, numerous efforts have been made to develop efficient electrocatalysts for the conversion of CO_2_, CO, N_2_, and H_2_O to valuable chemicals, such as hydrocarbons, oxygenates, and ammonia^4–12^. In particular, the electrochemical reduction of CO_2_ over Cu-based catalysts has received considerable interest, because Cu exhibits appreciable activity for C–C coupling to form multicarbon products, including ethylene, ethanol, and propanol^13,14^. While efforts are focused on developing catalytic materials, it is also critical to understand other factors beyond catalytic materials, such as the local environment of the catalysts^15^, which can mediate the transport and local concentration of reaction species and influence reaction pathways^16^.

**Fig. 1:**
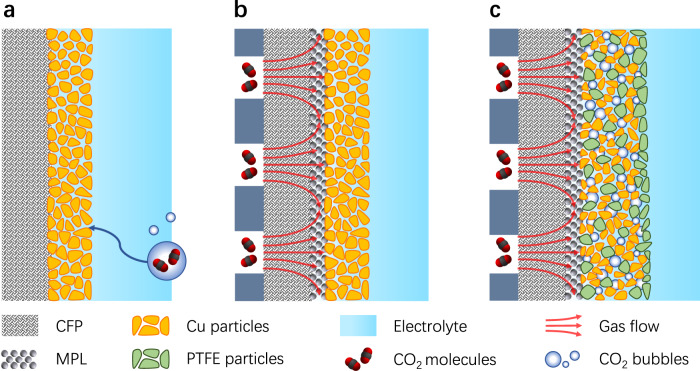
Schematic illustration of different catalyst microenvironments and reaction interfaces. **a** Solid–liquid interface in an H-cell. **b** Solid–liquid interface in a regular GDE cell. **c** Proposed hydrophobic microenvironment with solid–liquid–gas interfaces that can be constructed in a GDE cell by dispersing PTFE nanoparticles inside the catalyst layer.

Electrochemical CO_2_ reduction reaction (CO_2_RR) has been typically evaluated using H-type cells (H-cells)^6–8^, where the electrode is immersed in liquid electrolyte, and CO_2_ molecules dissolve in the electrolyte and diffuse down a concentration gradient to the catalyst surface for reactions^9^, as schematically shown in Fig. 1a. While this cell configuration works well for evaluating CO_2_RR at low current densities^6–10^, the low solubility and slow diffusion of CO_2_ in the electrolyte will cause a mass transport limitation at high current densities. The limiting current density for CO_2_RR on a planar electrode can be estimated by: *j*_lim_ = *nFD*_0_*C*_0_/*δ*, where *n* is the number of electrons transferred in the reaction, *F* is the Faraday constant, *D*_0_ and *C*_0_ are the diffusion coefficient and solubility of CO_2_ in the electrolyte, and *δ* is the diffusion layer thickness. The diffusion layer is a virtual layer of the CO_2_ concentration gradient interval^17^, which extends from the electrode surface to the point where the concentration of CO_2_ reaches the bulk concentration, as illustrated in Supplementary Fig. 1. Typically, the diffusion layer thickness is of the order of magnitude of 100 μm for CO_2_RR in H-cell^18^, resulting in a limiting current density of the order of 10 mA cm^−2^, as indicated by the estimation in Supplementary Fig. 1.

To alleviate the limitations of mass transport, flow cells with gas-diffusion electrodes (GDEs) have been developed and used to investigate electrochemical CO_2_ or CO reduction^19–28^. A GDE typically consists of a carbon fiber paper (CFP), a microporous layer (MPL), and a catalyst layer^20^. The catalyst side of a GDE is in contact with the electrolyte and the other side is exposed to flowing reactant gas, which diffuses through the pores in the CFP to reach the catalyst, as schematically illustrated in Fig. 1b. The MPL is composed of carbon powder and polytetrafluoroethylene (PTFE) particles, which can maintain the separation of the liquid and gas phases to prevent flooding of the pores in the CFP^20^. The catalyst particles in a GDE are often wetted by electrolyte due to their lack of hydrophobicity, as a result the reaction occurs primarily in aqueous phase via dissolved CO_2_ (refs. ^29–31^). In this cell configuration, reactant molecules diffuse through a relatively thin layer of electrolyte to reach the catalyst^29,30^, which greatly reduces the diffusion layer thickness and enables high-rate CO_2_ electrolysis at current densities >200 mA cm^−2^, as indicated by the plot in Supplementary Fig. 1. Nevertheless, the catalyst layer typically has a thickness of at least a few micrometers^18^, so the CO_2_RR may still be limited by CO_2_ mass transport inside the three-dimensional catalyst layer^32^.

Furthermore, the greatly improved CO_2_RR performance in GDE cells was also attributed to local gaseous environment and three-phase interfaces between solid catalyst, liquid electrolyte, and gaseous CO_2_ in some studies^21,22^. However, such argument remains under debate, that is, whether the CO_2_RR in a GDE cell can occur at a solid–liquid–gas interface via gaseous CO_2_, in contrast to the conventional electrode–electrolyte interface^29^. Recently, a few studies explored the three-phase interfaces for CO_2_ or CO reduction in H-cells^33–38^, typically using a hydrophobic substrate for the electrode. Although the electrode was immersed in liquid electrolyte in an H-cell, the hydrophobic substrate might trap gaseous reactant near the catalyst layer to change the local environment and form solid–liquid–gas interfaces, which could improve the activity and selectivity for CO_2_ or CO reduction^34–36^. These studies revealed the significant impact of the local gas/liquid environment of the catalysts in gas-involving electrochemical reactions^38^. However, much remains to be understood regarding the catalyst microenvironment and reaction interfaces, such as how to create an optimal microenvironment with solid–liquid–gas interfaces, and how such an environment affects the mass transport and kinetics of electrocatalytic reactions.

Here, we present a study of a hydrophobic microenvironment with solid–liquid–gas interfaces for gas-involving electrocatalysis, particularly CO_2_ reduction on Cu catalyst. As a proof-of-concept, we select commercially available Cu nanoparticles as the catalyst, so that the conclusions do not rely on any specially designed catalyst and can be generally applicable. We first show that using a hydrophobic substrate for the electrode improves the activity and selectivity for CO_2_RR in H-cell, validating the impact of the local environment. Then we design a GDE with a hydrophobic catalyst microenvironment for CO_2_ gas-diffusion electrolysis by dispersing PTFE nanoparticles in the catalyst layer, where the hydrophobic PTFE can repel liquid electrolyte and maintain gaseous reactant near the catalyst particles, as schematically shown in Fig. 1c. As a result, this electrode shows a significant improvement in the activity and Faradaic efficiency for CO_2_RR as compared to regular GDEs without added PTFE. The improved catalytic performance is attributed to a balanced gas/liquid microenvironment that reduces the diffusion layer thickness, and enhances the mass transport and kinetics of CO_2_ electrolysis, providing a general approach to improve gas-involving electrocatalysis.

## Results

### Characterization of Cu nanocatalyst

Commercial Cu nanoparticles (see Supplementary Note 1) were used as the electrocatalyst for CO_2_RR in this study. The Cu catalyst is less active than those specially designed Cu catalysts^19–22^, but it is widely available and often used as a reference sample in CO_2_RR studies^19,21^. The nanoparticles were characterized by transmission electron microscopy (TEM), X-ray diffraction (XRD), and X-ray photoelectron spectroscopy (XPS) to examine their size and composition, as presented in Supplementary Fig. 2. The TEM images and derived particle size distribution revealed an average size of 47.9 ± 16.8 nm of the Cu nanoparticles. XRD pattern showed diffraction peaks of Cu and a small fraction of Cu_2_O, of which the latter was due to oxidation by air. XPS survey spectrum showed mainly Cu and O peaks, where the O was attributed to the Cu_2_O component. To further identify the chemical state of the Cu catalyst during CO_2_RR, operando X-ray absorption spectroscopy (XAS) characterization was performed, as shown in Supplementary Fig. 3, and the acquired Cu K-edge XAS spectra indicated that the catalyst was reduced to metallic Cu state under CO_2_RR conditions^39^.

### Microenvironment for CO_2_RR in H-cell

**Fig. 2:**
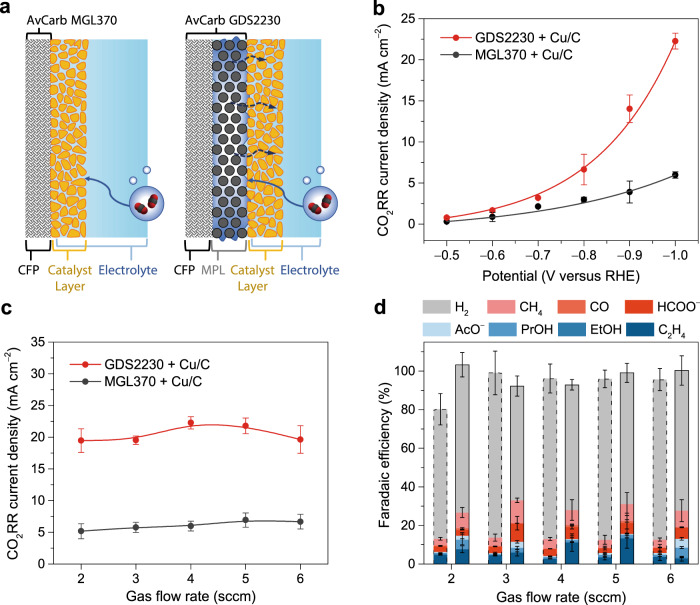
Effect of hydrophobic substrate on the CO_2_RR in H-cell. **a** Configurations of the two electrodes prepared with AvCarb MGL370 and GDS2230 substrates. **b** Partial current densities for CO_2_RR on the two electrodes at various potentials with a CO_2_ gas flow rate of 4 sccm. **c** Partial current densities and **d** Faradaic efficiencies for CO_2_RR on the two electrodes at −1.0 V with various CO_2_ flow rates. In **d**, the left column with dashed line frame at each flow rate is for the AvCarb MGL370 + Cu/C electrode, and the right column with solid line frame is for the AvCarb GDS2230 + Cu/C electrode. The error bars represent the standard deviation of three independent measurements.

We first examined the microenvironment for CO_2_RR on the Cu catalyst in H-cell, where a simple model of solid–liquid interface can be used to describe the reaction interface (Fig. 1a). To probe the effect of substrate hydrophobicity on the electrode performance, two substrates purchased from the Fuel Cell Store were used for comparison: AvCarb MGL370 CFP, and AvCarb GDS2230 consisting of CFP and a hydrophobic MPL coating. Contact angle measurements on them (Supplementary Fig. 4) revealed superior hydrophobicity of the AvCarb GDS2230 (151.7°) relative to the MGL370 (119.0°). Electrodes were prepared by depositing the catalyst ink (a mixture of Cu nanoparticles and carbon black) on the two substrates, and their configurations are schematically shown in Fig. 2a. Scanning electron microscopy (SEM) images suggested that the morphology of the catalyst layers on the two substrates was very similar (Supplementary Fig. 5). CO_2_RR tests were performed in an H-cell with CO_2_ gas bubbling into the cathodic compartment (Supplementary Fig. 6). The CO_2_RR performance was evaluated by controlled potential electrolysis in 1 M KHCO_3_ electrolyte. All potentials were reported with respect to the reversible hydrogen electrode (RHE) in this study. Gas-phase products were quantified by periodic gas chromatography, and solution-phase products were analyzed at the end of each electrolysis by nuclear magnetic resonance (NMR) spectroscopy (Supplementary Fig. 7).

CO_2_RR was first evaluated at various potentials ranging from −0.5 to −1.0 V vs RHE for both electrodes, and representative chronoamperometric curves are shown in Supplementary Fig. 8. As expected, the partial current density for CO_2_RR increased exponentially with the overpotential for both electrodes (Fig. 2b). Interestingly, the CO_2_RR current densities on the GDS2230 electrode were generally higher than that on the MGL370 electrode, particularly at higher overpotentials. For example, a CO_2_RR current density of ~23 mA cm^−2^ was reached at −1.0 V on the GDS2230 electrode, which is about four times that on the MGL370 electrode (~6 mA cm^−2^). As there was no major difference between the two electrodes regarding the morphology (Supplementary Fig. 5) or the conductivity (as revealed by the electrochemical impedance spectra (EIS) in Supplementary Fig. 9), their difference in CO_2_RR performance is attributed to the substrate hydrophobicity, most likely because the hydrophobic MPL can repel liquid electrolyte and trap gas bubbles^40,41^.

To verify the liquid repelling effect of the MPL, we measured the contact angles of the AvCarb MGL370 and GDS2230 substrates (no catalyst loading) after electrochemical treatment at −1.0 V in the electrolyte. As shown in Supplementary Fig. 4, the contact angle of the MGL370 substrate dropped significantly from 119.0° to 22.5° due to electrochemical modifications. In contrast, the GDS2230 substrate remained similarly hydrophobic with a contact angle of ~150° after the treatment, so the MPL cannot be wetted or flooded by the electrolyte and gas bubbles can be maintained in the pores of the MPL. When the GDS2230 electrode is tested for CO_2_RR, the gas bubbles trapped inside the MPL can serve as an intermediate reservoir of gaseous CO_2_ for the reaction. Thus, the diffusion layer thickness decreases to the distance between the gas bubbles in the MPL and the catalyst particles^42^, which improves CO_2_ mass transport to the catalyst layer and increases the CO_2_RR limiting current density. This is also supported by the potential-dependent difference in the CO_2_RR performance between the two electrodes: the CO_2_RR current density was similar at −0.5 V, but the difference was enlarged to 4-fold at −1.0 V where the CO_2_RR became limited by mass transport (Fig. 2b), confirming that CO_2_ mass transport in the GDS2230 electrode is improved by the MPL. This mechanism can also explain the enhanced performance for CO reduction on hydrophobic electrodes^35,36^.

How is gaseous CO_2_ formed in the MPL? It can be formed directly by trapping the purged CO_2_ gas bubbles^34^, or indirectly from the dissolved CO_2_ molecules in the electrolyte^43^. If it is the former case, the gas bubbling rate will affect the trapping of gaseous CO_2_ and the CO_2_RR rate^34^; otherwise the CO_2_RR rate should not depend on the gas bubbling rate in the latter case, as long as the electrolyte remains saturated with CO_2_. In the H-cell, the electrode is positioned ~1 cm away from the gas inlet (Supplementary Fig. 6), so it is less likely to directly trap gas bubbles. We varied the CO_2_ gas bubbling rate to examine the gas trapping by the MPL. Figure 2c shows the CO_2_RR current density measured on the two electrodes at −1.0 V with various CO_2_ gas bubbling rates, ranging from 2 to 6 standard cubic centimeters per minute (sccm). Both current densities remained largely unchanged with the bubbling rate, indicating that the CO_2_RR mainly relied on the dissolved CO_2_ molecules for both electrodes. We postulate that the hydrophobic MPL can facilitate the nucleation and formation of CO_2_ gas bubbles from the CO_2_-saturated electrolyte^44^. Similarly, the Faradaic efficiency for CO_2_RR also showed a weak dependence on the gas flow rate (Fig. 2d). The total Faradaic efficiency for CO_2_RR on the GDS2230 and MGL370 electrodes was ~30% and 13%, respectively. We attribute the difference to a higher local concentration of CO_2_ due to the improved mass transport by the MPL^34^. The difference in the CO_2_RR selectivity confirmed the impact of the electrode hydrophobicity and corresponding local environment on the CO_2_RR.

### Hydrophobic microenvironment for CO_2_RR in GDE cell

It was shown above that a hydrophobic substrate can change the local gas/liquid environment and improve the mass transport for CO_2_RR in an H-cell. In a GDE cell, the catalyst layer typically has a thickness of at least a few micrometers^18,32^, so the MPL is unlikely to influence the microenvironment deep inside the catalyst layer. Therefore, we designed an electrode with local hydrophobic centers by dispersing PTFE particles inside the catalyst layer, where the PTFE can repel liquid electrolyte and maintain gas bubbles in neighboring pores, as schematically shown in Fig. 1c. In particular, PTFE nanoparticles of 30−40 nm in size (Nanoshel LLC) were used, which have a similar size as the Cu nanoparticles and can enable a uniform mixing, as verified by the energy-dispersive X-ray spectroscopy (EDS) elemental mapping in Supplementary Fig. 10. Thus, the PTFE nanoparticles can trap numerous gas bubbles in the catalyst layer and enforce a high surface area gas–liquid interface near the catalyst particles during CO_2_RR.

**Fig. 3:**
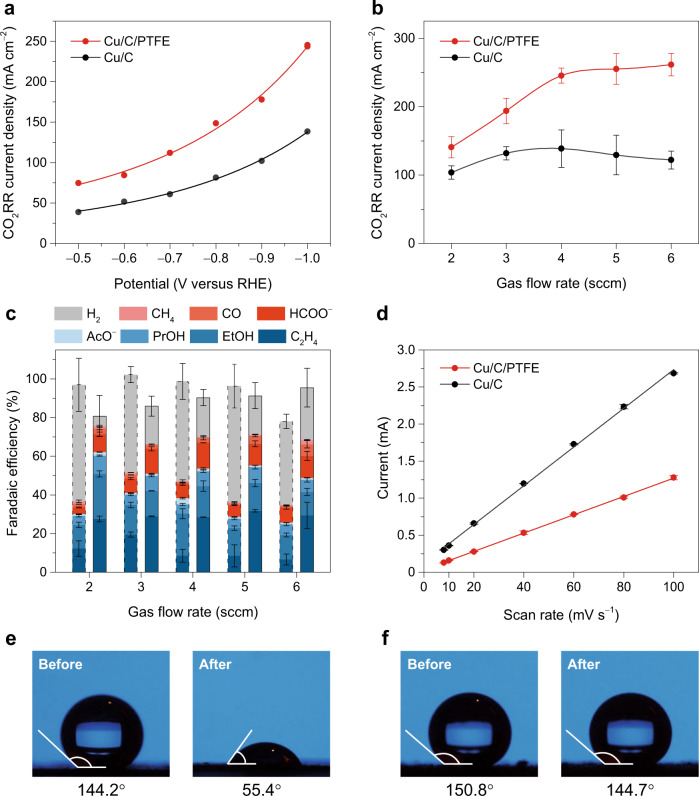
Effect of hydrophobic microenvironment on the CO_2_RR in GDE cell. **a** Partial current densities for CO_2_RR on the Cu/C and Cu/C/PTFE electrodes at various potentials with a CO_2_ gas flow rate of 4 sccm. **b** Partial current densities and **c** Faradaic efficiencies for CO_2_RR on the two electrodes at −1.0 V with various CO_2_ flow rates. In **c**, the left column with dashed line frame at each flow rate is for the Cu/C electrode and the right column with solid line frame is for the Cu/C/PTFE electrode. **d** Double-layer charging current plotted against the CV scan rate for the two electrodes. **e**, **f** Photographs of contact angle measurements on the **e** Cu/C electrode and **f** Cu/C/PTFE electrode before and after CO_2_ electrolysis at −1.0 V for 2 h. The error bars represent the standard deviation of three independent measurements.

To understand the effect of the hydrophobic microenvironment, two electrodes were prepared for comparison: one using the original catalyst ink (Cu nanoparticles and carbon black), and the other using PTFE-dispersed catalyst ink with a 50% mass ratio of PTFE, both deposited on the AvCarb GDS2230 substrate. The two electrodes have the same loading of Cu nanoparticles, and they are referred as Cu/C and Cu/C/PTFE electrodes, respectively. SEM images indicated that the morphology of the catalyst layers of the two electrodes was very similar (Supplementary Fig. 11). CO_2_ gas-diffusion electrolysis was tested using a home-built GDE flow cell (Supplementary Fig. 12) with circulating 1 M KOH electrolyte (Supplementary Fig. 13). The electrodes were first evaluated at various potentials, ranging from −0.5 to −1.0 V. As shown in Fig. 3a, the partial current density for CO_2_RR on the Cu/C electrode increased from 39 mA cm^−2^ at −0.5 V to 138 mA cm^−2^ at −1.0 V, much higher than that measured for the same electrode in the H-cell (Fig. 2b). The Cu/C/PTFE electrode showed an even higher CO_2_RR current density than the Cu/C electrode at each potential. Particularly, a partial current density of ~250 mA cm^−2^ was reached for CO_2_RR on the Cu/C/PTFE electrode at −1.0 V, which was almost twice that of the Cu/C electrode. We postulate that the dispersed PTFE nanoparticles in the catalyst layer form hydrophobic gas channels, which reduce the electrolyte layer thickness that CO_2_ must diffuse from the point of dissolution to the catalyst surface. This greatly decreases the diffusion layer thickness for the catalyst particles inside the catalyst layer, thus improving the CO_2_ mass transport and CO_2_RR performance.

To distinguish if the CO_2_ transport inside the catalyst layer was mainly mediated by gas-phase or aqueous-phase diffusion, we compared the CO_2_RR activity on the two electrodes with various CO_2_ gas flow rates. As presented in Fig. 3b, the CO_2_RR current density on the Cu/C electrode at −1.0 V showed a weak dependence on the flow rate, which increased from 104 to 138 mA cm^−2^ as the flow rate increased from 2 to 4 sccm, but declined to 122 mA cm^−2^ at a flow rate of 6 sccm. In contrast, the CO_2_RR current density on the Cu/C/PTFE electrode showed a distinct trend, which increased almost linearly from 140 to 250 mA cm^−2^ as the flow rate increased from 2 to 4 sccm and then continued to increase mildly at higher flow rates. As a result, a maximum single-pass conversion rate of 14% was reached for CO_2_RR on the Cu/C/PTFE electrode at 4 sccm, which is about twice that of the Cu/C electrode (7.3%) at the same flow rate (Supplementary Fig. 14). As previously discussed, if the CO_2_RR is only mediated by aqueous-phase transport of dissolved CO_2_ molecules to the catalyst, the reaction rate should not be affected by the CO_2_ gas flow rate (Fig. 2c). Here, the strong dependence of the CO_2_RR current density on the flow rate for the Cu/C/PTFE electrode indicated a gas-phase transport of CO_2_ in the catalyst layer via hydrophobic channels. In addition, the CO_2_RR selectivity was different between the two electrodes, as presented in Fig. 3c. The total Faradaic efficiency for CO_2_RR on the Cu/C electrode ranged between 35 and 50% at various flow rates, while the total Faradaic efficiency on the Cu/C/PTFE electrode was higher, ranging between 68 and 76%. The Faradic efficiency for C_2+_ products was also higher on the Cu/C/PTFE electrode, suggesting that the electrode increased the local concentration of the intermediate product CO and consequently enhanced the C–C coupling process^16,45^.

It is noted that the added PTFE will increase the catalyst layer thickness of the Cu/C/PTFE electrode, which can influence the diffusion of CO_2_ and CO_2_RR activity. As revealed by the SEM images in Supplementary Fig. 15, the catalyst layer thickness was estimated to be 23.5 ± 2.1 and 39.3 ± 2.6 μm for the Cu/C and Cu/C/PTFE electrodes, respectively. To evaluate the influence of the catalyst layer thickness, an additional Cu/C electrode with extra carbon black loading was prepared (referred as Cu/C-extra electrode), of which the catalyst layer thickness (40.6 ± 1.8 μm) is close to that of the Cu/C/PTFE electrode. A comparison of their CO_2_RR performance was shown in Supplementary Fig. 15d: the partial current density and total Faradaic efficiency for CO_2_RR on the Cu/C-extra electrode was similar to that of the Cu/C electrode, but the Faradaic efficiency for C_2+_ products was lower on the Cu/C-extra electrode, which was attributed to the relatively lower concentration of CO_2_ inside the catalyst layer^16^. This is reasonable as CO_2_ needs to diffuse over a longer distance on average to reach the catalyst particles in a thicker catalyst layer. Interestingly, the Cu/C/PTFE electrode had a similarly thicker catalyst layer, but its CO_2_RR current density and C_2+_ Faradaic efficiency were both much higher than that of the Cu/C and Cu/C-extra electrodes, confirming the improvement of CO_2_ mass transport and CO_2_RR performance by the hydrophobic microenvironment, despite a thicker catalyst layer.

To further verify the presence of gaseous reactant inside the catalyst layer, we compared the electrochemically active surface area (ECSA) of the two electrodes. ECSA represents the area of an electrode that is wetted and accessible to the electrolyte. We postulate that the increased volume of gas within the catalyst layer will reduce its ECSA due to less contact with the electrolyte. The ECSA is proportional to the electrochemical double-layer capacitance, which can be measured by cyclic voltammetry (CV) in a potential window where only double-layer charging and discharging occur^46^, as illustrated in Supplementary Fig. 16. The double-layer charging current was plotted against the scan rate, and the slope of the linear regression gives the double-layer capacitance. As shown in Fig. 3d, the capacitance of the Cu/C/PTFE electrode (~12.4 mF) was around half that of the Cu/C electrode (~26.1 mF), despite the same loading of Cu and carbon black. This confirmed the presence of gas bubbles in the catalyst layer and the formation of solid–liquid–gas interfaces.

A balance between gas and liquid in a GDE may be broken during electrolysis, as the electrode often becomes hydrophilic due to electrochemical modifications so that the pores in the catalyst layer are flooded by the electrolyte^30,31^, which will suppress the mass transport and lead to a decline of the reaction rate. For example, as shown in Fig. 3e, the catalyst side of the Cu/C electrode exhibited a contact angle of 144.2° initially, which however dropped significantly to 55.4° after CO_2_RR at −1.0 V for 2 h, indicating an evolution of the electrode’s hydrophobicity and flooding of the electrode^31^. In contrast, the Cu/C/PTFE electrode exhibited a contact angle of 150.8° and 144.7° before and after electrolysis (Fig. 3f), suggesting that the added PTFE particles preserved the hydrophobicity and prevented the catalyst layer from flooding, so that a balanced gas/liquid microenvironment was maintained in the catalyst layer to form durable solid–liquid–gas interfaces for CO_2_ electrolysis.

### Effects of PTFE loading and size on the microenvironment

**Fig. 4:**
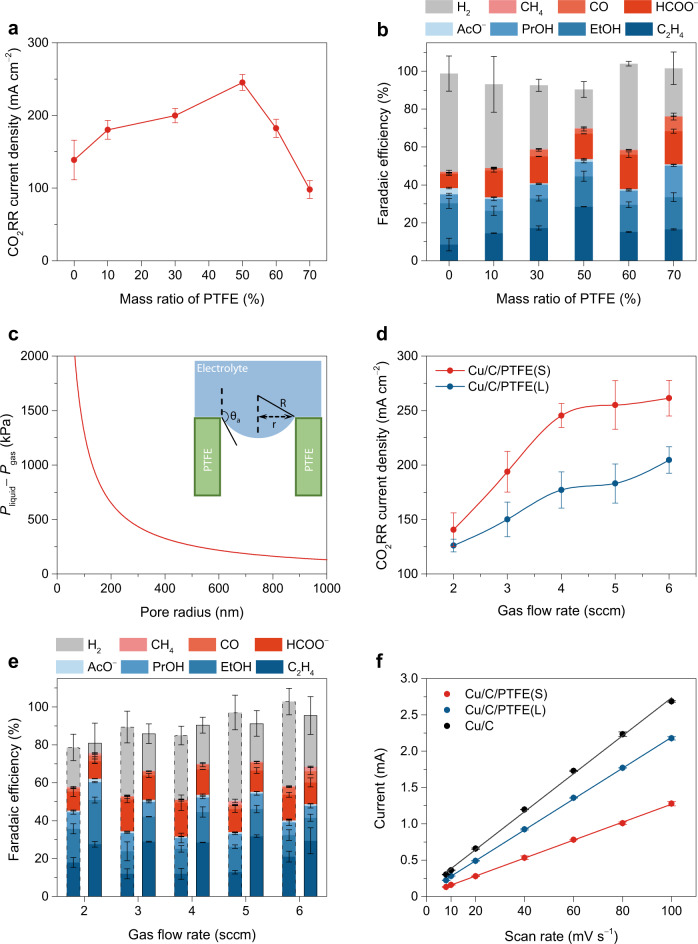
Effects of PTFE loading and size on the microenvironment for CO_2_RR in GDE cell. **a** Partial current densities and **b** Faradaic efficiencies for CO_2_RR at −1.0 V on Cu/C/PTFE electrodes with different mass ratios of PTFE in the catalyst layer. **c** Pressure difference sustained across the liquid–gas interface in nano-sized pores as a function of the pore radius. Inset: schematic of the interface advancing inside a pore. **d** Partial current densities and **e** Faradaic efficiencies for CO_2_RR on two Cu/C/PTFE electrodes with different PTFE particle sizes. In **e**, the left column with dashed line frame at each flow rate is for the Cu/C/PTFE(L) electrode and the right column with solid line frame is for the Cu/C/PTFE(S) electrode. **f** Double-layer charging current plotted against the CV scan rate for the electrodes. The error bars represent the standard deviation of three independent measurements.

The gas/liquid microenvironment inside the catalyst layer depends on the added PTFE particles, particularly their loading and size. To elucidate their effects, we first varied the loading of the PTFE nanoparticles with otherwise the same amount of Cu nanoparticles and carbon black. Figure 4a shows the partial current densities for CO_2_RR on the Cu/C/PTFE electrodes with different PTFE mass ratios in the catalyst layer. As the mass ratio increased from 0, the CO_2_RR activity increased until a maximum value was reached at a 50% mass ratio of PTFE, while an even higher ratio caused a decline of the activity. The total Faradaic efficiency for CO_2_RR exhibited a similar dependence on the PTFE mass ratio from 0 to 50%, but it did not drop at a higher ratio of 70% (Fig. 4b). Thus, a moderate amount of PTFE can create a hydrophobic microenvironment to enhance the CO_2_RR activity and Faradaic efficiency, but excessive PTFE will over suppress the availability of electrolyte and protons for CO_2_RR. An optimal balance between gas and liquid in the catalyst layer is needed for efficient CO_2_ electrolysis. To directly build a relationship between the electrode hydrophobicity and CO_2_RR performance, we measured the contact angles of these electrodes and plotted the CO_2_RR current density versus the contact angles, as shown in Supplementary Fig. 17. The contact angles before CO_2_RR were close, ranging from 144.2° (0% PTFE) to 155.1° (70% PTFE), but the contact angles after CO_2_RR decreased to various degrees: the more the PTFE loading was, the larger the contact angle remained. Therefore, only the contact angle measured after electrolysis reflects an electrode’s capability of repelling liquid and stabilizing gas/liquid microenvironment in the catalyst layer for CO_2_RR.

The gas–liquid balance in the catalyst layer also depends on the size of hydrophobic pores, which is correlated with the PTFE particle size. The capillary pressure difference sustained across the interface between liquid and gas is determined by the Young–Laplace equation: *P*_liquid_ − *P*_gas_ = 2*σ*/*R*, where *σ* is the surface tension of 1 M KOH electrolyte (74.4 mN m^−1^)^47^, and *R* is the radius of curvature of the interface. In addition, as illustrated in Fig. 4c, *R* = *r*/sin(*θ*_a_ − 90*°*), where *r* is the pore radius and *θ*_a_ is the advancing contact angle of the electrolyte on the electrode (~150.8*°*). Based on the equation, a smaller pore requires a higher critical burst-through pressure for liquid to enter the pore^33^, as plotted in Fig. 4c. Thus, the catalyst layer with smaller PTFE particles should form smaller hydrophobic pores that are more effective in repelling liquid and maintaining gas in the pores.

To verify the effect of PTFE particle size, two Cu/C/PTFE electrodes were prepared with different PTFE particles: one is of 30−40 nm in size (Nanoshel LLC), and the other is of ~1 μm in size (Sigma Aldrich), both with a 50% mass ratio in the catalyst layer. They are referred as Cu/C/PTFE(S) and Cu/C/PTFE(L), respectively. The two electrodes were evaluated for CO_2_RR in the GDE cell with various CO_2_ flow rates. As shown in Fig. 4d, the CO_2_RR current density on the Cu/C/PTFE(L) electrode similarly increased with the flow rate, but it was generally lower than that on the Cu/C/PTFE(S) electrode due to the larger hydrophobic pores with a weaker repelling of liquid electrolyte. Similar difference was observed in the total Faradaic efficiency for CO_2_RR on the two electrodes, as well as the Faradic efficiency for C_2+_ products, as shown in Fig. 4e. The effect of PTFE particle size on the formed microenvironment can be further examined by double-layer capacitance that reflects the area wetted by the electrolyte. The linear fit in Fig. 4f revealed a capacitance of 21.2 mF of the Cu/C/PTFE(L) electrode, which is larger than that of the Cu/C/PTFE(S) electrode (~12.4 mF), but still smaller than that of the Cu/C electrode (~26.1 mF), validating the effect of PTFE particle size in creating a gas/liquid microenvironment inside the catalyst layer.

### Effect of gas-diffusion channels

**Fig. 5:**
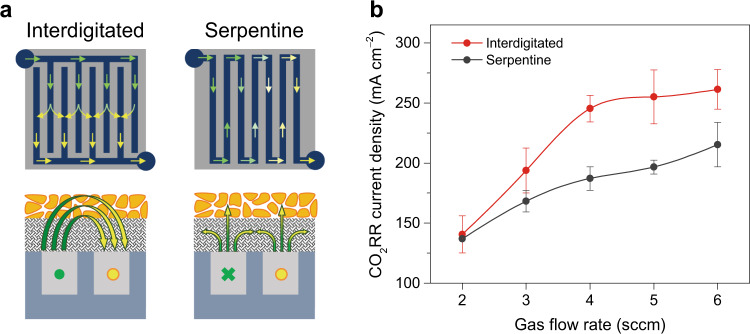
Effect of gas-diffusion channels on the CO_2_RR in GDE cell. **a** Schematic illustration of the gas flow fields generated by the interdigitated and serpentine channels. **b** Partial current densities for CO_2_RR on the Cu/C/PTFE electrode (with a 50% PTFE mass ratio) with two different channels at −1.0 V with various CO_2_ gas flow rates. The error bars represent the standard deviation of three independent measurements.

As the CO_2_RR activity of the Cu/C/PTFE electrode depends on the CO_2_ gas flow rate, the gas flow field in the GDE can be engineered to enhance the CO_2_ transport via the design of gas-diffusion channels, such as interdigitated and serpentine channels (Fig. 5a). Recent studies of CO_2_RR in GDE cells often used serpentine channels^21,24^, where the neighboring channels are connected so that gas diffuses along the channels from inlet to outlet. In this design, the vertical diffusion of gas into the electrode and catalyst layer is driven by pressure gradient. In contrast, in the interdigitated design the inlet and outlet rows are aligned alternately and separately by walls, so the inlet gas is forced to diffuse vertically into the electrode and then exit to the outlet channels^48^. Such a flow field is more effective in driving the mass transport of CO_2_ into the catalyst layer. Thus, we compared CO_2_RR on the Cu/C/PTFE electrode in GDE cells with interdigitated and serpentine flow fields. As shown in Fig. 5b, the CO_2_RR current density increased with the gas flow rate for both designs, but the one with interdigitated channels showed a higher CO_2_RR current density, as well as a sharper increase with the flow rate, indicating a more efficient transport of gaseous CO_2_ to the catalyst with the interdigitated flow field.

## Discussion

The above results confirmed the formation of a balanced gas/liquid microenvironment inside the catalyst layer of the Cu/C/PTFE electrode for CO_2_ electrolysis. Compared to regular GDEs, the added PTFE particles create hydrophobic pores for gas-phase CO_2_ transport inside the catalyst layer, which greatly reduces the diffusion layer thickness as compared to a regular catalyst layer that is wetted by electrolyte^29^. To quantify the effect, we obtained the EIS of the electrodes under CO_2_RR conditions and model them to estimate the diffusion layer thickness. EIS is an effective and noninvasive method to investigate Nernst diffusion process in a multilayer system^49^, which can be described by an equivalent impedance *Z*_d_ in circuit modeling. Supplementary Fig. 18 shows the circuit model and its equivalent ladder circuit to describe the impedances in a porous carbon electrode^50^, as well as the EIS measured for the Cu/C, Cu/C/PTFE(S), and Cu/C/PTFE(L) electrodes under CO_2_RR conditions in the GDE cell. The feature in the low frequency region of the EIS is attributed to the impedance of the diffusion layer^49^. The finite diffusion layer thickness *δ* in our system can be theoretically derived from *Z*_d_ (ref. ^51^), and the diffusion layer thickness was estimated to be 20.2 ± 3.1, 3.2 ± 0.9, and 7.3 ± 0.8 μm for the Cu/C, Cu/C/PTFE(S), and Cu/C/PTFE(L) electrodes, respectively. The data further quantitatively confirmed our conclusion: the diffusion layer thickness greatly reduced from 20.2 to 3.2 μm after dispersing PTFE particles in the catalyst layer, because the PTFE can enable gas-phase transport of CO_2_ in the catalyst layer and reduce the thickness of liquid electrolyte that CO_2_ must diffuse through to reach the catalyst. The estimated diffusion layer thickness for the Cu/C/PTFE(L) electrode is also consistent with our expectation and the measured CO_2_RR performance.

The reduced diffusion layer thickness accelerates the transport of CO_2_ to the catalyst, resulting in an increased steady concentration of CO_2_ near the catalyst^29^, as shown in Supplementary Fig. 19. At equilibrium, the surface coverage of *CO_2_ ($\theta_{{\mathrm{CO}}_2}$) adsorbed on the catalyst is proportional to the local concentration of CO_2_ as follows^16^: $\theta_{{\mathrm{CO}}_2}$ = θ*·[CO_2_]·exp(−$E_{{\mathrm{CO}}_2}$/*RT*), where θ* is the coverage of available surface sites, [CO_2_] is the CO_2_ local concentration, $E_{{\mathrm{CO}}_2}$ is the adsorption energy of CO_2_ on the catalyst, *R* is the ideal gas constant, and *T* is the temperature. Therefore, we propose that the hydrophobic microenvironment in the Cu/C/PTFE electrode enhances the mass transport and adsorption of CO_2_, resulting in an increased coverage of *CO_2_ on the catalyst surface for reactions^18,32^. This will increase the CO_2_RR rate, as well as the produced CO for C–C coupling, thus improving the Faradaic efficiency for C_2+_ products. The hydrophobic microenvironment may also trap the produced CO inside the catalyst layer, which can increase the local concentration of CO to enhance C–C coupling toward C_2+_ products^16,45^.

Furthermore, the ECSA of the Cu/C/PTFE electrode is around half that of the Cu/C electrode (Fig. 3d), so half of the Cu nanoparticles are not in contact with the electrolyte and the surfaces of these catalyst particles are inactive for CO_2_RR due to the lack of protons and ionic conductivity. On the other hand, some catalyst particles may be located at the boundary between gas and liquid, so they are accessible to both gaseous CO_2_ molecules and liquid electrolyte. Thus, CO_2_ molecules from the gas side, and protons or water molecules from the liquid side can promote CO_2_RR at the three-phase boundary sites of the catalyst surface, as schematically shown in Supplementary Fig. 20. Such three-phase boundary sites can be highly active for CO_2_RR due to the direct and fast gas-phase adsorption of CO_2_ on the catalyst surface without the influence of electric double layer and solvated ions^52–54^. This can explain the dependence of the CO_2_RR activity on the gas flow rate for the Cu/C/PTFE electrode due to the gas-phase transport and adsorption of CO_2_.

As a proof-of-concept study, we used commercial Cu nanoparticles for simplicity, which are intrinsically less active than those optimized Cu catalysts^19–22^. As a result, the CO_2_RR performance here may not be as high as that in some reports, but the significance of our study lies in the new understanding and a general approach to control the catalyst microenvironment for gas-involving electrochemical reactions. Our work differs from some prior studies that tuned the composition of the MPL^20^ or added PTFE suspensions in the catalyst layer^55^, where the PTFE particles were coated by surfactant that could weaken the hydrophobicity. In addition, some studies used CO_2_ flow rates as high as 50 or 100 sccm^21,24^, which may create a high local pressure in the gas side of the GDE^56^ and enhance the CO_2_ mass transport to improve CO_2_RR performance^57^. Our study achieved a high activity and selectivity for CO_2_RR with a much lower CO_2_ flow rate (4 sccm), resulting in a high single-pass conversion rate of CO_2_ of ~14% (Supplementary Fig. 14), benefiting from the catalyst microenvironment. Our method of controlling local gas/liquid microenvironment can be generally applied to improve other gas-involving electrocatalysis, when gaseous reactant has a low solubility and slow diffusion in the electrolyte, such as the electrochemical reduction of N_2_ (ref. ^58^).

In summary, we developed a GDE with a hydrophobic microenvironment for CO_2_ electrolysis by dispersing PTFE nanoparticles in the catalyst layer, where the PTFE can repel liquid electrolyte and maintain gaseous reactant near the catalyst particles. The Cu/C/PTFE electrode showed a significant improvement in the activity, Faradaic efficiency, and C_2+_ product selectivity for CO_2_RR as compared to a regular Cu/C electrode without added PTFE. Furthermore, the CO_2_RR current density on the Cu/C/PTFE electrode increased with the CO_2_ gas flow rate, indicating a gas-phase transport of CO_2_ in the catalyst layer. The improved performance is attributed to the reduced diffusion layer thickness that accelerates CO_2_ mass transport, increases the local concentration of CO_2_ near the catalyst surface, and enhances CO_2_ adsorption for the reaction. Compared to regular GDEs, the electrode with added PTFE particles creates a balanced gas/liquid microenvironment and solid–liquid–gas interfaces inside the catalyst layer, which can enhance the mass transport and kinetics of CO_2_ electrolysis, providing a general approach to improve gas-involving electrocatalysis.

## Methods

### Materials characterization

TEM images were acquired using a FEI Tecnai F30 transmission electron microscope with a field emission gun operated at 200 kV. SEM images and EDS elemental mapping were acquired using a ZEISS Ultra-55 FEG scanning electron microscope. XRD pattern was collected using a PANalytical Empyrean diffractometer with a 1.8 KW copper X-ray tube. XPS data were acquired by a Thermo Scientific ESCALAB XI^+^ X-ray Photoelectron Spectrometer with an Al Kα X-ray source (1486.67 eV). Operando XAS was performed at Beamline 2-2 of the Stanford Synchrotron Radiation Lightsource at the SLAC National Accelerator Laboratory using a modified two-compartment H-cell and a Lytle fluorescence detector (Supplementary Fig. 3). The XAS data were processed using the ATHENA software^59^. Contact angle measurements were carried out using an L2004A1 Ossila Contact Angle Goniometer (Ossila Ltd, UK).

### Preparation of electrodes for CO_2_RR in H-cell

First, 6 mg of commercial Cu nanoparticles (US1828, US Research Nanomaterials) and 6 mg of Vulcan XC 72 carbon black (Fuel Cell Store) were each dispersed in 2 mL isopropanol, respectively. After sonication for 1 h, the two dispersions were mixed with 200 μL Nafion solution (5 wt%) and sonicated for another 1 h. The mixture was used as the catalyst ink and sprayed on electrode substrates by a homemade XY plotter equipped with an airbrush. Two types of substrates with an area of 1 × 1 cm^2^ were used: AvCarb MGL370 and AvCarb GDS2230 (Fuel Cell Store). After deposition, the electrodes were dried overnight at room temperature, with a Cu catalyst loading of 0.65 ± 0.05 mg cm^−2^.

### Preparation of electrodes for CO_2_RR in GDE cell

The same catalyst ink in the H-cell studies was used as 0% PTFE-catalyst ink here. The PTFE-dispersed catalyst layer was prepared as follows. First, 6 mg of commercial Cu nanoparticles (US1828, US Research Nanomaterials) and 6 mg of Vulcan XC 72 carbon black (Fuel Cell Store) were each dispersed in 1 mL isopropanol, respectively. Then, 2.2, 8.7, 20, 30, and 46.7 mg PTFE nanopowder (APS 30−40 nm, Nanoshel LLC) were dispersed in 2 mL isopropanol, respectively. After sonication for 1 h, Cu nanoparticle dispersion, carbon black dispersion, corresponding PTFE dispersion, and 200 μL Nafion solution (5 wt%, containing ~8 mg Nafion) were mixed and sonicated for another 1 h, which were used as 10%, 30%, 50%, 60%, and 70% PTFE-catalyst inks, respectively. Each catalyst ink was sprayed on an AvCarb GDS2230 substrate with a Cu catalyst loading of 0.65 ± 0.05 mg cm^−2^. After drying overnight, 1 mL of diluted Teflon PTFE DISP 30 solution (0.12 wt%, Fuel Cell Store) was further sprayed on top of all electrodes except the 0% PTFE one. All the samples were dried in air for at least 5 h before testing.

### Electrochemical measurements

Electrochemical tests were performed using a Gamry Interface 1000 Potentiostat or a CH Instruments 760E Potentiostat with an H-cell or a home-built GDE flow cell. The H-cell experiments were carried out in a gas-tight two-compartment H-cell separated by a Nafion 1110 membrane under ambient conditions (Supplementary Fig. 6). A platinum gauze and an Ag/AgCl electrode with saturated KCl solution (BASi MF-2056) were used as the counter electrode and the reference electrode, respectively. Electrodes prepared with AvCarb MGL370 or GDS2230 substrate were used as the working electrode. CO_2_-saturated 1 M KHCO_3_ solution was used as the electrolyte, which was stirred at a rate of 600 r.p.m. during electrolysis. GDE-cell studies were performed using a home-built GDE flow cell (Supplementary Fig. 12), including a Ti current collector with interdigitated gas-diffusion channels, a cathodic GDE with catalyst layer on AvCarb GDS2230 substrate, a 3D-printed chamber with ports for electrolyte flow and reference electrode, and an Fe–Ni foam inserted in a pocket of Ti current collector as the anode^28^. The gas-diffusion channels have a depth of 0.2 mm and a density of 50 channels cm^−1^. A Nafion 1110 or FAA-3-PK-130 membrane was used to separate the cathode and anode chambers. A leak-free Ag/AgCl electrode (Warner Instruments) was used as the reference electrode. The above prepared electrodes were used as working electrodes with an effective area of 0.66 cm^2^. The catholyte and the anolyte were each 20 mL of 1 M KOH solution circulated using peristaltic pumps at a flow rate ranging from 0.6−2.2 mL min^−1^. For both H-cell and GDE-cell studies, CO_2_ gas flow was controlled by an Alicat mass flow controller at a specified flow rate ranging from 2−6 sccm, and the applied potentials were iR-compensated and converted to the RHE scale. The reported partial current densities for CO_2_RR were normalized to geometric surface areas. The EIS data were fit with a circuit model^50^ using the EIS Spectrum Analyser^60^.

During electrolysis, gas-phase products from the H-cell or GDE cell were quantified by a gas chromatograph (SRI Multiple Gas Analyzer #5) equipped with molecular sieve 5A and HayeSep D columns with Ar as the carrier gas. Solution-phase products were analyzed using a Bruker AVIII 500 MHz NMR spectrometer. Typically, 500 µL of the post-electrolysis catholyte was mixed with 100 µL of D_2_O containing 100 p.p.m. dimethyl sulfoxide as the internal standard. ^1^H NMR spectra were acquired using water suppression mode.

### Electrochemically active surface area measurement

The ECSA of an electrode was quantified by measuring the double-layer capacitance. CV was performed in the GDE flow cell at different scan rates in a potential window where only double-layer charging and discharging occur (no Faradaic process). The double-layer charging current was then plotted versus the CV scan rate, and the slope of the linear regression gave the double-layer capacitance. A representative set of the CV scans is exhibited in Supplementary Fig. 16.

### Calculation of CO_2_RR current density and Faradaic efficiency

The gas-phase products were quantified by comparison of the peak integrals to standard calibration gases to determine the molar flow rate of a product (*V*). The Faradaic efficiency (FE) for each gas-phase product was calculated using the following equation:

$${\mathrm{FE}} = \frac{{nFV}}{{I_{{\mathrm{total}}}}} \times 100\%,$$

where *n* is the number of electrons transferred for the product, *F* is the Faraday constant, *V* is the molar flow rate of the product, and *I*_total_ is the total current of the electrolysis. The molar quantities of solution-phase products were quantified by NMR spectroscopy and then converted to Coulombs by multiplying by *nF*, where *F* is Faraday’s constant and *n* = 2, 8, 12, and 18 for formate (HCOO^−^), acetate (AcO^−^), ethanol (EtOH), and *n*-propanol (PrOH), respectively. The charges corresponding to each product were then compared to the integrated electrolysis charge to determine the Faradaic efficiency.

The partial current density for CO_2_RR ($j_{{\mathrm{CO}}_{2}{\mathrm{RR}}}$) was calculated using the following equation:

$$j_{{\mathrm{CO}}_2{\mathrm{RR}}} = \frac{{\mathop {\sum }\nolimits_{{\mathrm{CO}}_2{\mathrm{RR}}\,{\mathrm{products}}} \left( {I_{{\mathrm{total}}} \times {\mathrm{FE}}} \right)}}{{{\mathrm{Electrode}}\,{\mathrm{area}}}},$$

where FE is the Faradaic efficiency of each product, and electrode area is the effective geometrical area of the working electrode.

The single-pass conversion rate (CR) of CO_2_ was calculated using the following equation:

$${\mathrm{CR}} = \frac{{\mathop {\sum }\nolimits_{{\mathrm{CO}}_2{\mathrm{RR}}\,{\mathrm{products}}} \left( {\frac{{I_{{\mathrm{total}}} \times {\mathrm{FE}}}}{{nF}} \times N_{\mathrm{C}} \times \frac{{RT}}{P}} \right)}}{{{\mathrm{CO}}_2\,{\mathrm{flow}}\,{\mathrm{rate}}}},$$

where *N*_C_ is the number of carbon atoms in each product molecule (for example, *N*_C_ = 2 for C_2_H_4_), *R* is the ideal gas constant, and *T* and *P* are the absolute temperature and pressure of the CO_2_ gas.

The reported CO_2_RR current densities, Faradaic efficiencies, conversion rates, and their error bars were determined based on the measurements of three separately prepared samples under the same conditions.

## Supplementary information

Supplementary Information

## Data Availability

The data that support the findings of this study are available in the article and its Supplementary Information file or from the corresponding authors upon reasonable request.
