# Supplementary material for: Enhancing carbon dioxide gas-diffusion electrolysis by creating a hydrophobic catalyst microenvironment
Source: Nat Commun. 2021 Jan 8;12:136. doi: 10.1038/s41467-020-20397-5 (PMC7794506; doi:10.1038/s41467-020-20397-5)
Supplement: Supplementary file 1 — Supplementary Information [file 41467_2020_20397_MOESM1_ESM.pdf]

Supplementary Information

Enhancing carbon dioxide gas-diffusion electrolysis by creating a hydrophobic catalyst microenvironment

Xing et al.

Supplementary Note 1

Chemicals:

Copper nanoparticles (Cu, 99.8%, US1828) were purchased from the US Research Nanomaterials, Inc. AvCarb MGL370 and AvCarb GDS2230 carbon substrates, Nafion 1110 and FAA-3-PK-130 membranes, carbon black (Vulcan XC-72), and Teflon PTFE DISP 30 Fluoropolymer Dispersion were purchased from the Fuel Cell Store. PTFE nanopowder (APS 30−40nm) was purchased from Nanoshel LLC. PTFE powder (1 μm particle size) and Nafion perfluorinated resin solution (5 wt%) were purchased from Sigma-Aldrich. Sulfuric acid (H_2SO_4, 98%), hydrogen peroxide (H_2O_2, 30%, Certified ACS), potassium bicarbonate ($KHCO_3$, 99.95%), potassium hydroxide (KOH, 99.98%), 2-propanol (Certified ACS), dimethyl sulfoxide (Certified ACS), deuterium oxide (D_2O, for NMR, 99.8 atom% D), and platinum gauze (99.9%, metal basis) were purchased from Fisher Scientific. Ag/AgCl reference electrode (MF-2056) was purchased from BASi. Leak-free Ag/AgCl electrode was purchased from Warner Instruments. CO_2 (99.999%), Ar (99.999%), and H_2 (99.999%) gases were purchased from Airgas. Deionized water with a specific resistance of 18.2 MΩ·cm was used throughout the experiments.

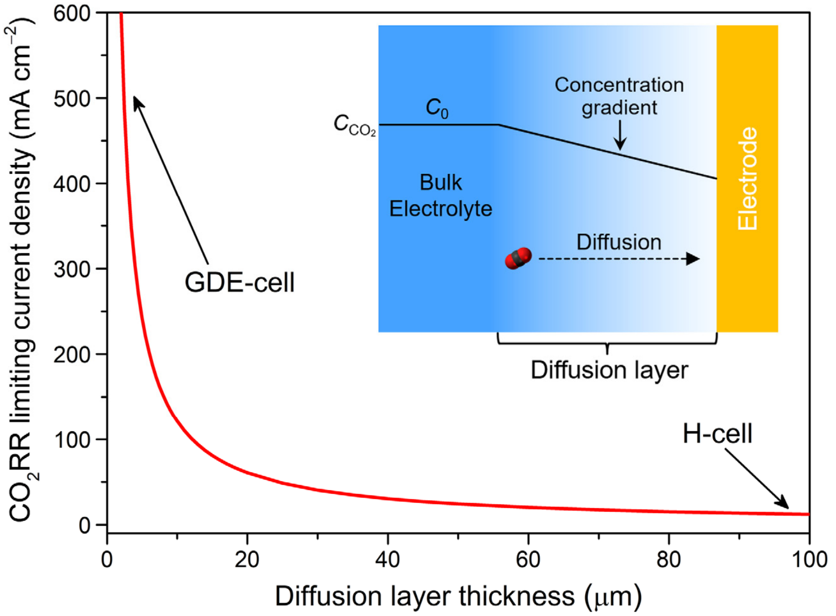

Supplementary Figure 1. Estimated limiting current density for CO_2RR on a planar electrode as a function of diffusion layer thickness based on equation: $j_{lim} = nFD_0C_0/\delta$, where n is the number of electrons transferred in the reaction (n = 2 for simplicity), F is the Faraday constant, D_0 and C_0 are the diffusion coefficient and solubility of CO_2 in the electrolyte ($D_0 = 1.91\times10^{-5}$ cm^2 s^{-1}, C_0 = 33 mM), and δ is the diffusion layer thickness. The inset shows a geometric model to explain the diffusion layer.

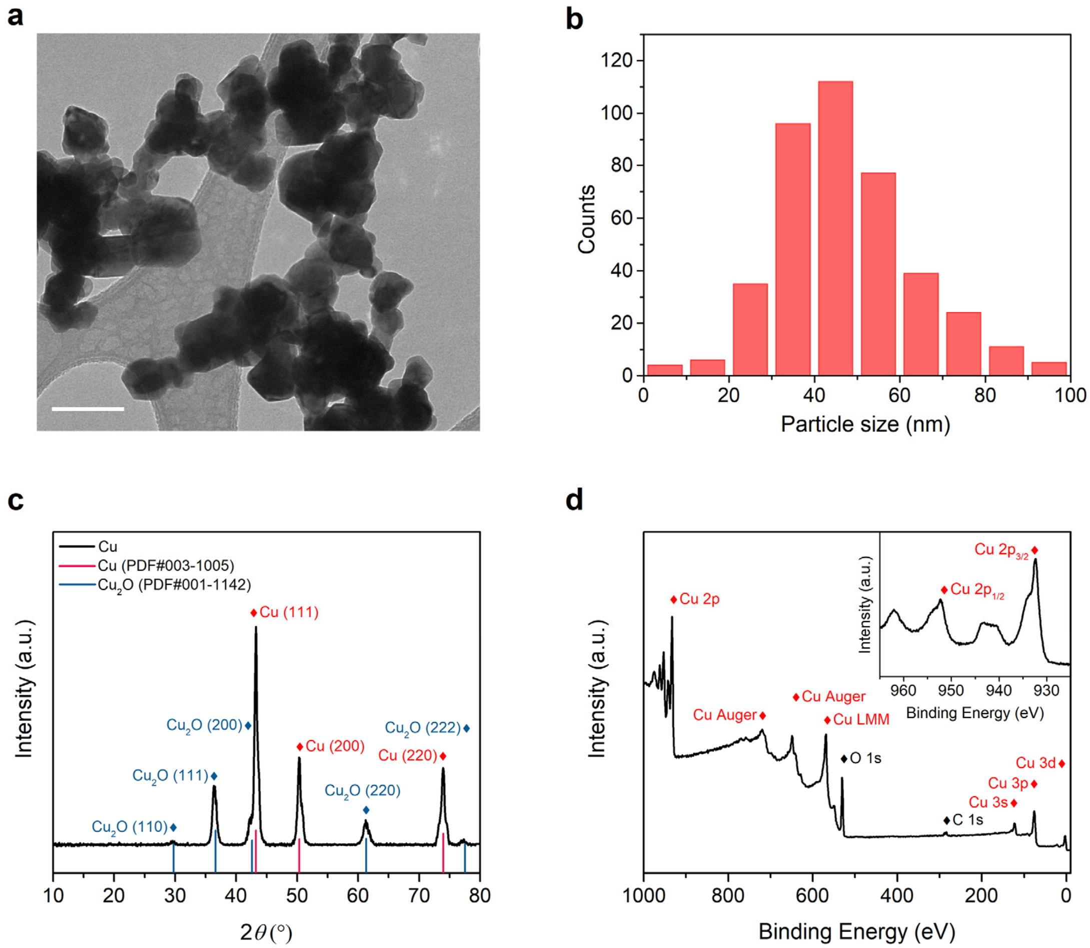

Supplementary Figure 2. Characterizations of the Cu nanoparticles. **a** A typical TEM image of the Cu nanoparticles. Scale bar: 100 nm. **b** Size distribution of the Cu nanoparticles derived from TEM images. **c** XRD pattern of the Cu nanoparticles. **d** XPS survey spectrum of the Cu sample. Inset: high-resolution XPS spectrum of Cu 2p region.

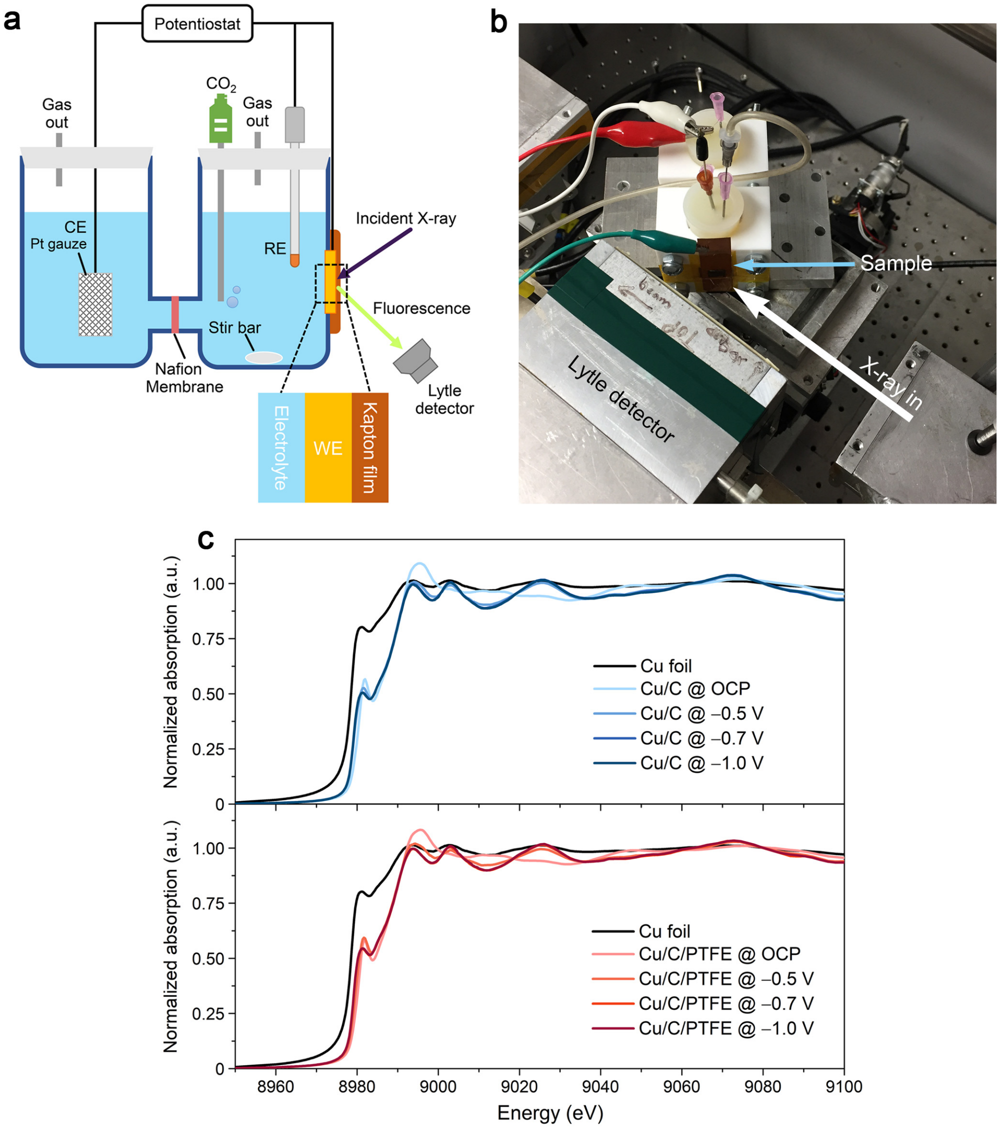

Supplementary Figure 3. *Operando* X-ray absorption spectroscopy (XAS) characterization of the Cu catalyst during CO_2RR. **a** Schematic illustration and **b** photograph of the modified H-cell setup for the XAS characterization. **c** Cu K-edge XAS spectra acquired over the Cu/C and Cu/C/PTFE electrodes in CO_2-satuated $KHCO_3$ electrolyte under different conditions, with the XAS spectrum of a Cu foil as a reference.

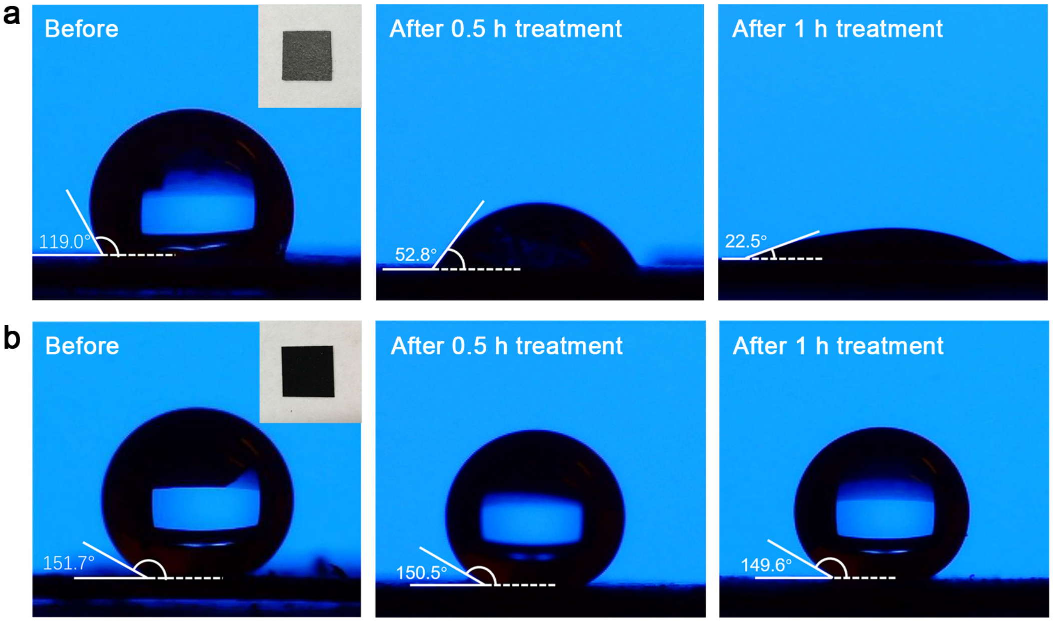

Supplementary Figure 4. Photographs of contact angle measurements on the **a** AvCarb MGL370 and **b** AvCarb GDS2230 substrates (no catalyst loading) before and after electrochemical treatment at −1.0 V vs RHE in 1 M $KHCO_3$ electrolyte. Insets: photograph of the corresponding substrate.

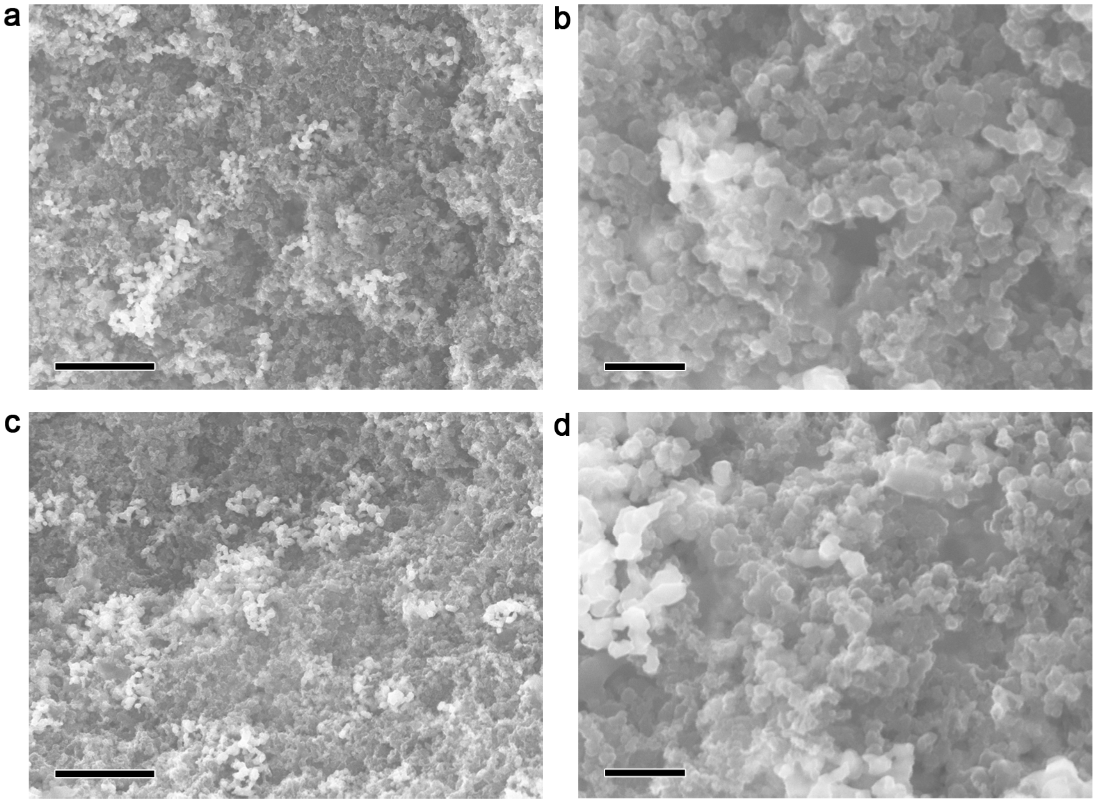

Supplementary Figure 5. Top-view SEM images of the **a, b** AvCarb MGL370 + Cu/C electrode; **c, d** AvCarb GDS2230 + Cu/C electrode. The scale bars are 2 μm in **a** and **c**, 400 nm in **b** and **d**.

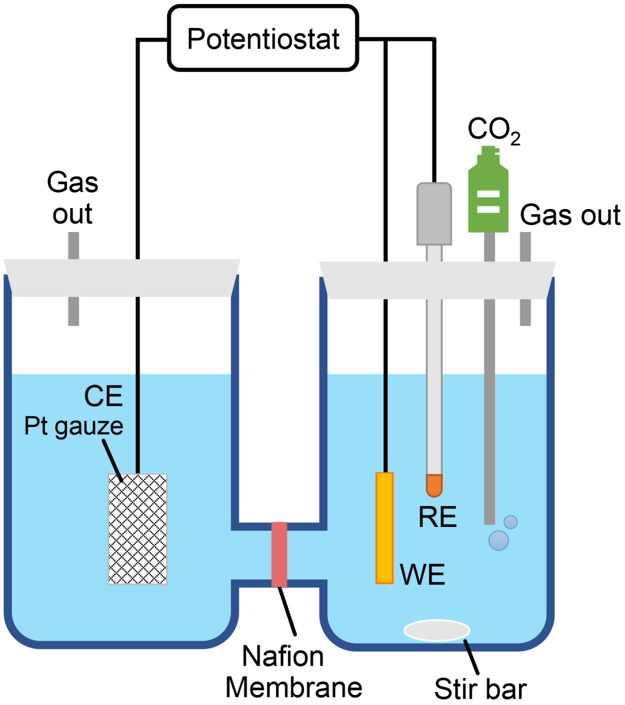

Supplementary Figure 6. Schematic diagram of the H-cell setup for CO_2RR measurements.

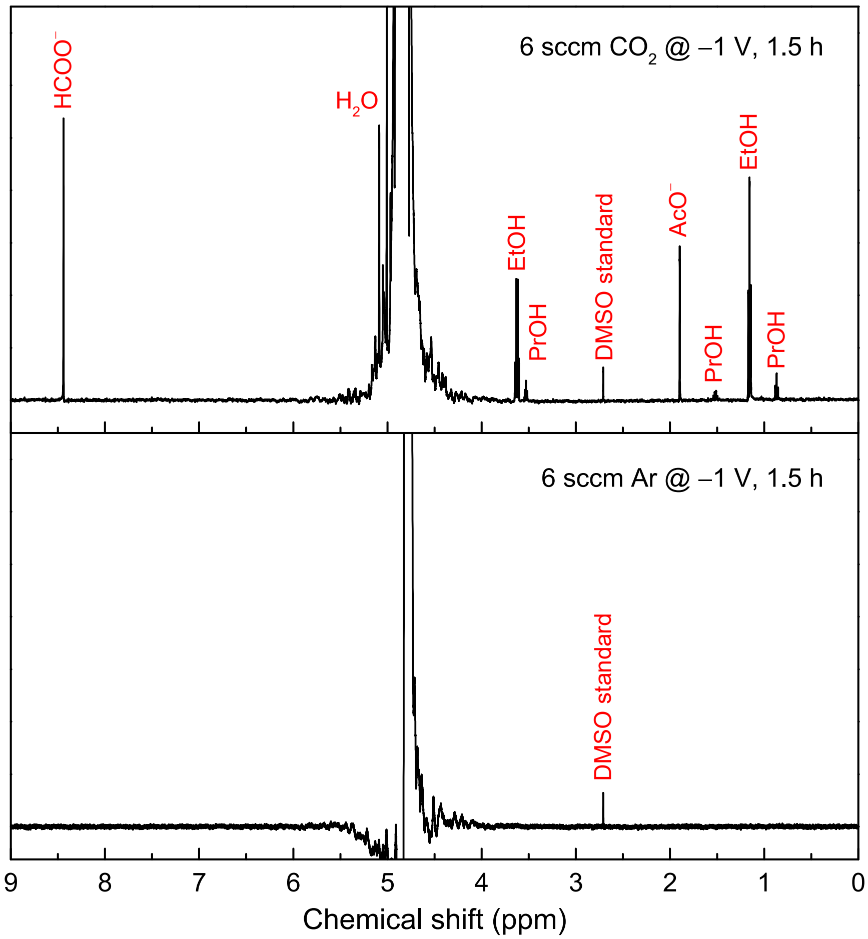

Supplementary Figure 7. Representative NMR spectra of the solution-phase products for CO_2RR over the Cu catalyst.

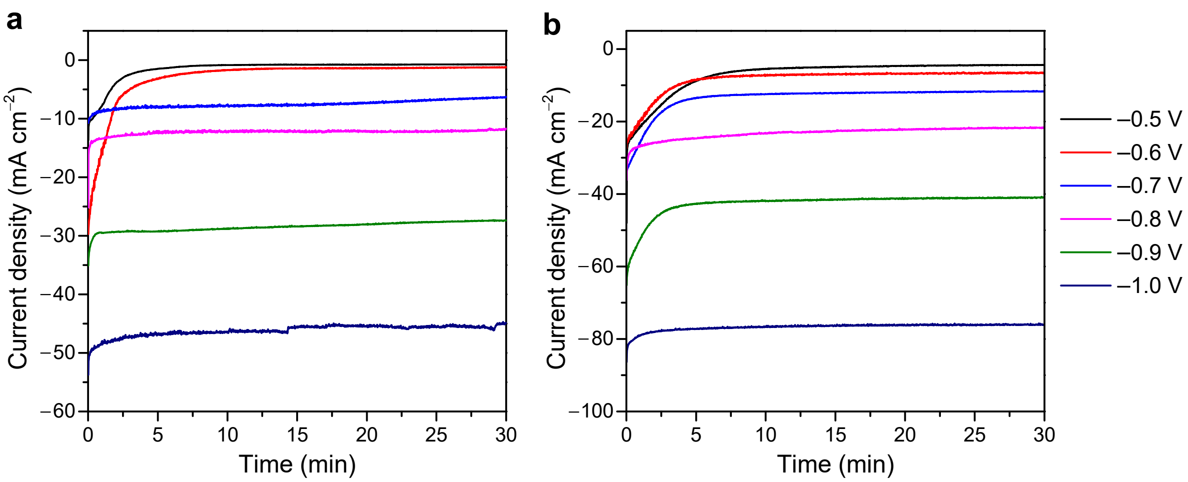

Supplementary Figure 8. Chronoamperometric curves for CO_2RR in the H-cell with 1 M $KHCO_3$ electrolyte at various potentials: **a** AvCarb MGL370 + Cu/C electrode; **b** AvCarb GDS2230 + Cu/C electrode.

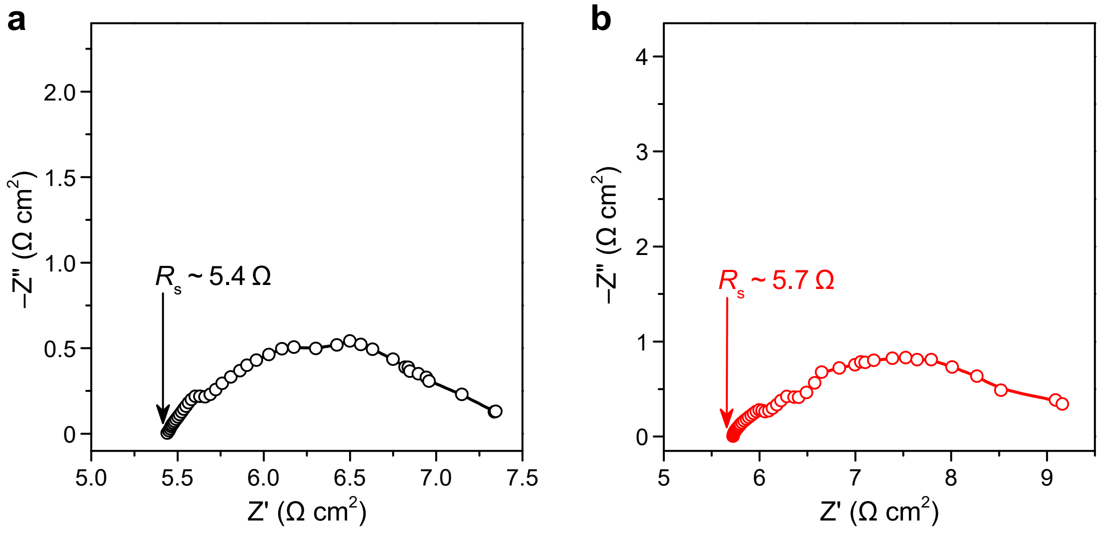

Supplementary Figure 9. Nyquist plots obtained using electrochemical impedance spectroscopy (EIS) for the **a** AvCarb MGL370 + Cu/C electrode and **b** AvCarb GDS2230 + Cu/C electrode under CO_2RR conditions at −1.0 V vs RHE in the H-cell. R_s indicates internal resistance.

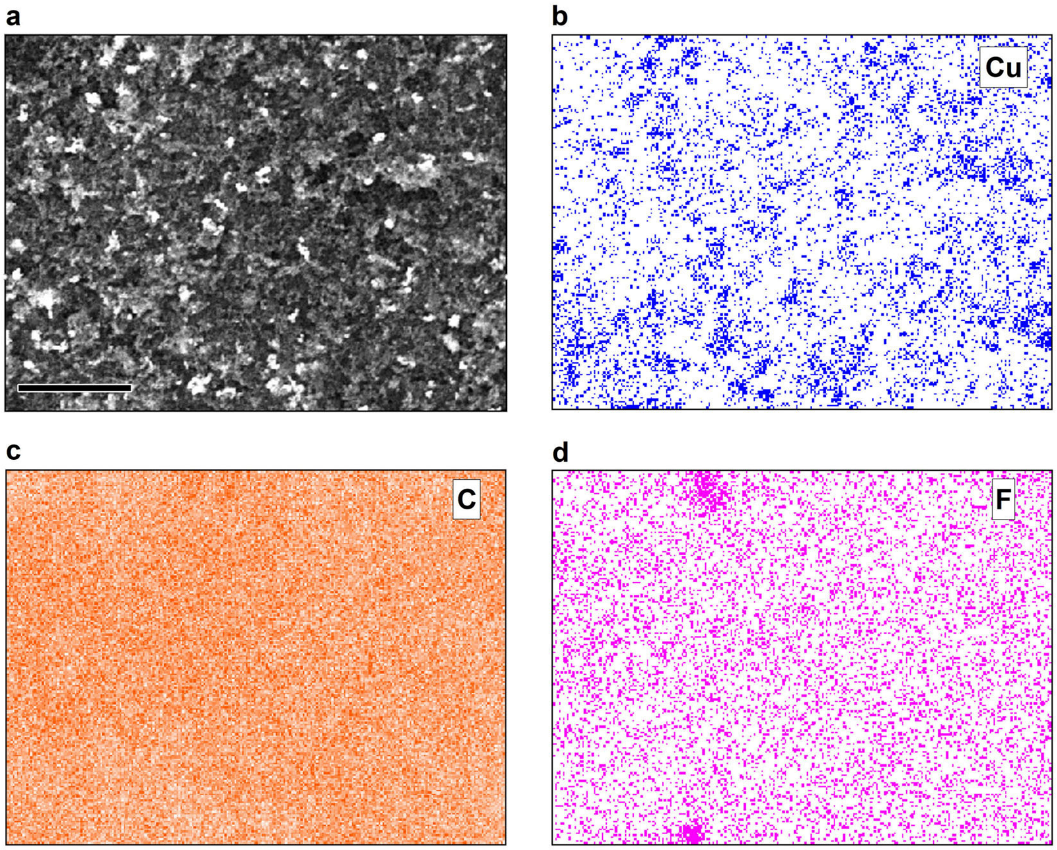

Supplementary Figure 10. a Top-view SEM image of AvCarb GDS2230 + Cu/C/PTFE electrode, and **b–d** corresponding energy-dispersive X-ray spectroscopy (EDS) mapping of Cu, C, and F elements in the same area. Scale bar: 5 μm.

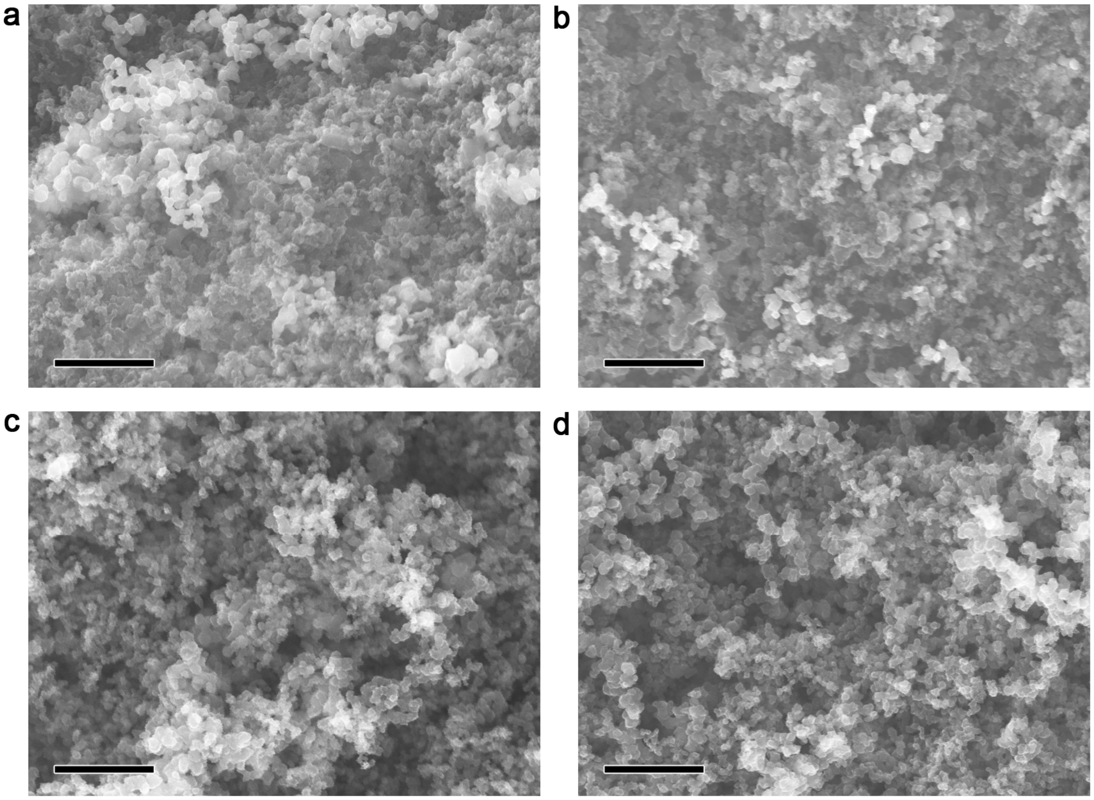

Supplementary Figure 11. Top-view SEM images of the Cu/C and Cu/C/PTFE electrodes on the AvCarb GDS2230 substrate before and after CO_2RR at −1.0 V vs RHE in the GDE-cell: **a** Cu/C and **b** Cu/C/PTFE electrodes before CO_2RR; **c** Cu/C and **d** Cu/C/PTFE electrodes after CO_2RR. Scale bars: 1 μm.

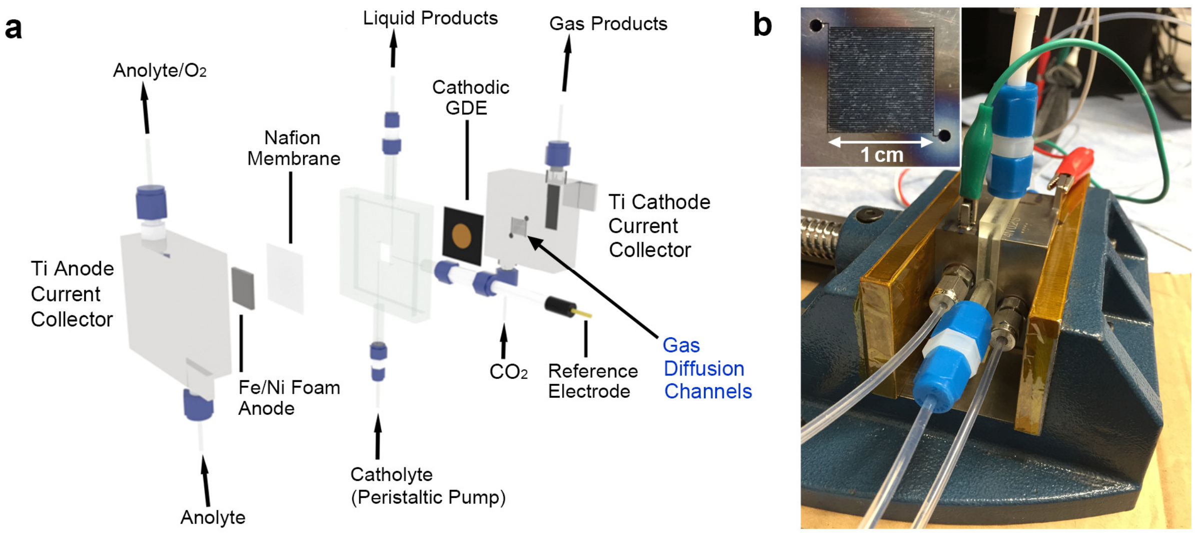

Supplementary Figure 12. a Schematic illustration showing the configuration of the GDE-cell for CO_2 electrolysis. **b** A photograph of the assembled GDE-cell setup, with the interdigitated gas-diffusion channels shown in the inset.

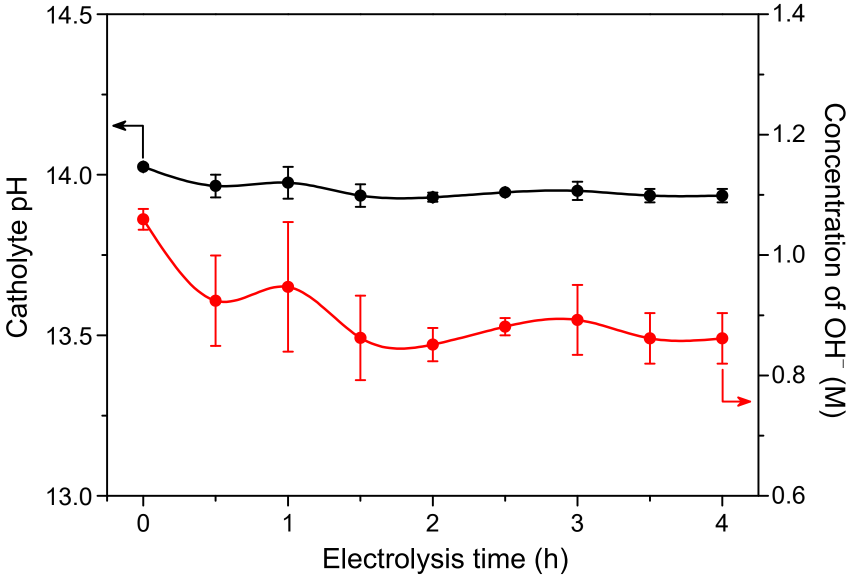

Supplementary Figure 13. Time-dependent pH of the 1 M KOH catholyte circulated during CO_2 electrolysis in the GDE flow cell. The decrease of pH was attributed to the reaction between CO_2 and OH^- in the electrolyte. The error bars represent the standard deviation of three independent measurements.

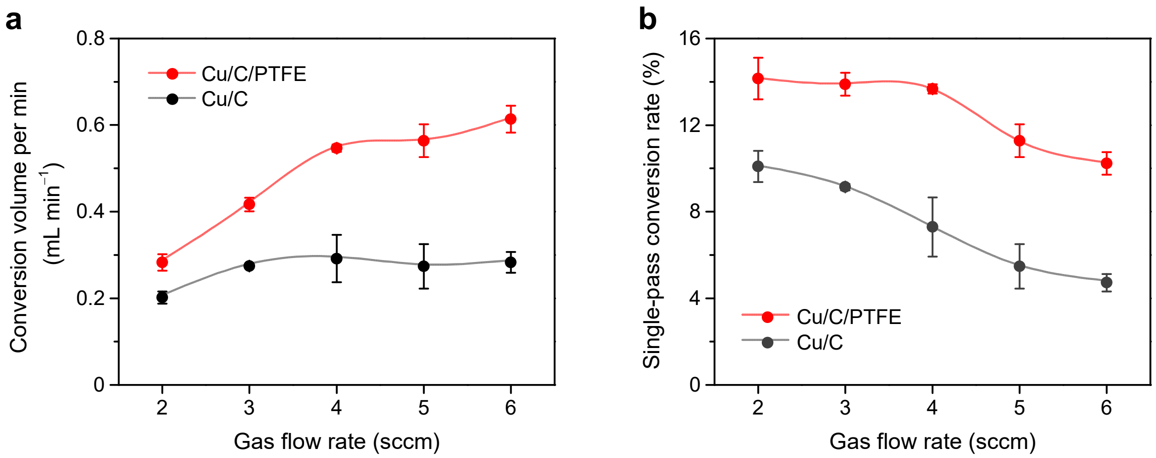

Supplementary Figure 14. a Single-pass conversion volume of CO_2 per min, and **b** single-pass conversion rate of CO_2 for the CO_2 electrolysis on the Cu/C and Cu/C/PTFE electrodes at −1.0 V vs RHE in the GDE-cell with various CO_2 gas flow rates. The error bars represent the standard deviation of three independent measurements.

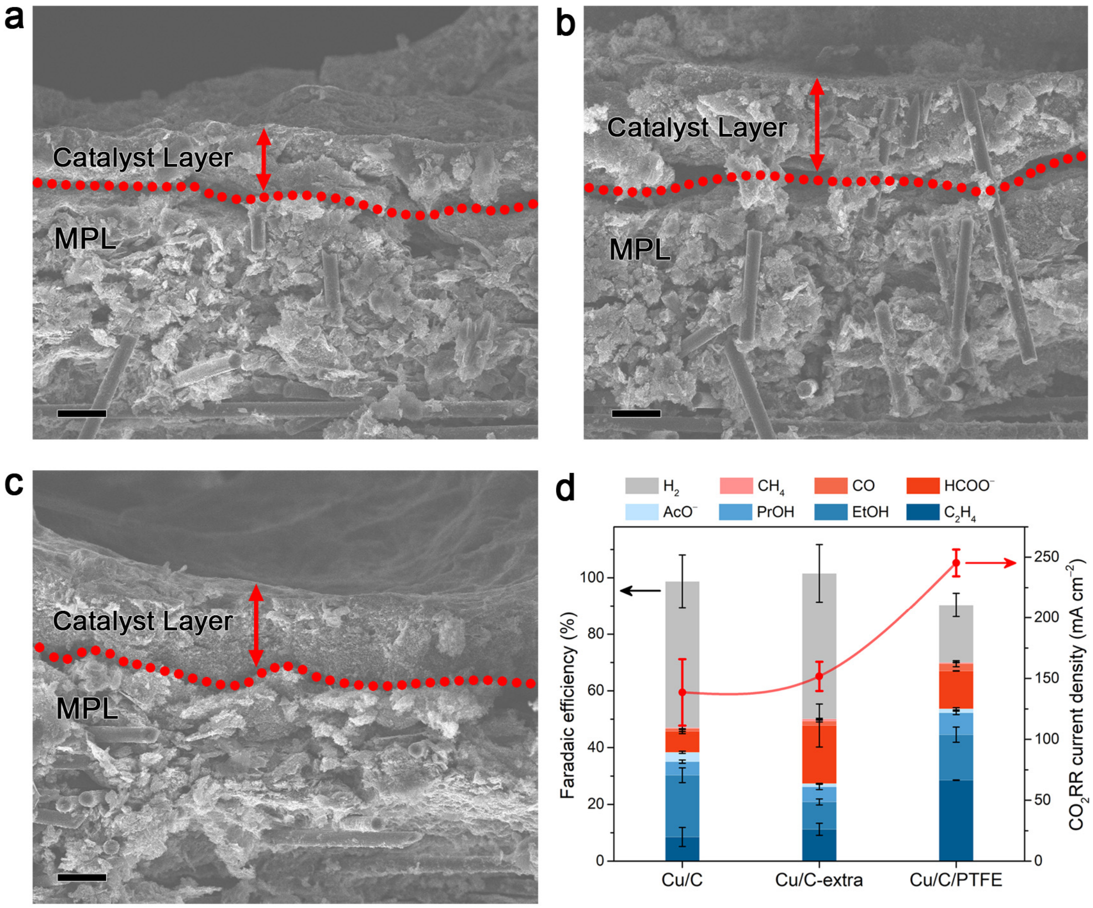

Supplementary Figure 15. Comparative experiment to evaluate the influence of the catalyst layer thickness. **a–c** Cross-sectional SEM images of the **a** Cu/C electrode; **b** Cu/C-extra electrode (extra loading of carbon black); **c** Cu/C/PTFE electrode. The boundary between the catalyst layer and the MPL is shown in each image. Scale bars: 20 μm. **d** Faradaic efficiency and partial current density for CO_2RR on the three electrodes at −1.0 V vs RHE in the GDE-cell. The error bars represent the standard deviation of three independent measurements.

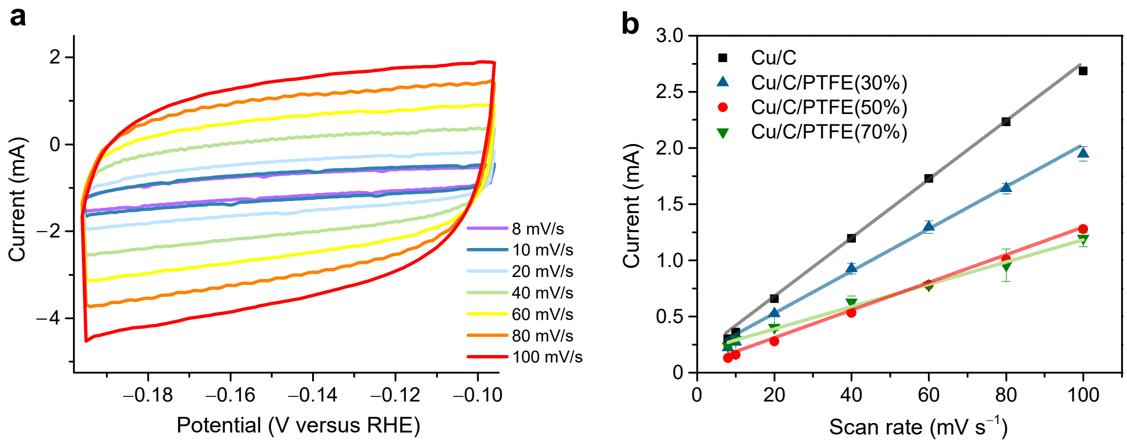

Supplementary Figure 16. Double-layer capacitance measurement. **a** Cyclic voltammagrams taken at various scan rates in a potential window where only double-layer charging and discharging occurs. **b** Double-layer charging current plotted against the scan rate for different electrodes. The slope of the linear regression line gives the double-layer capacitance. The error bars represent the standard deviation of three independent measurements.

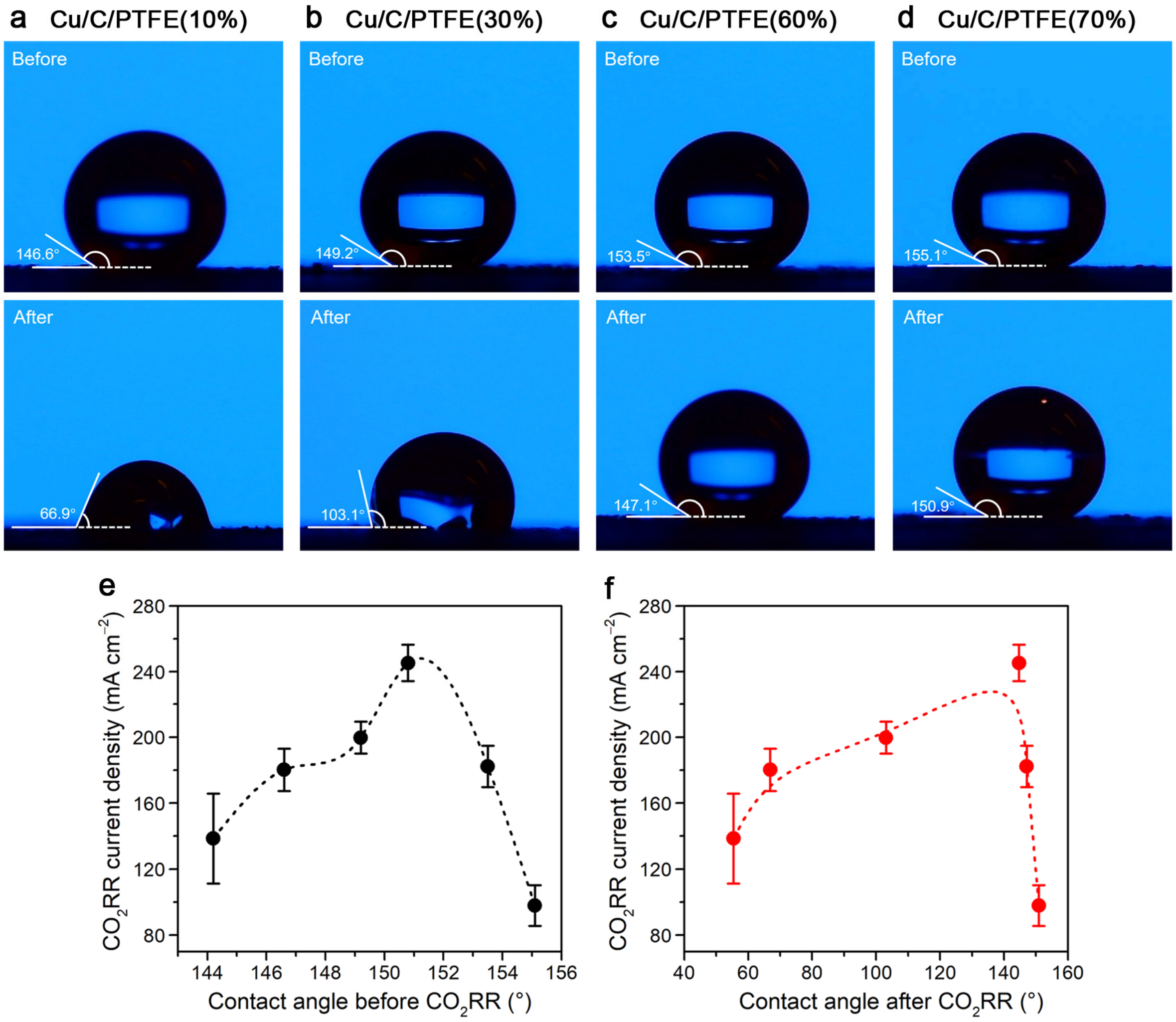

Supplementary Figure 17. Relationship between electrode wettability and CO_2RR performance. **a–d** Contact angle measurements on the Cu/C/PTFE electrodes with various PTFE loadings before and after CO_2RR at −1.0 V vs RHE in the GDE-cell. **e–f** Relationship between the CO_2RR current density and the contact angle of the electrodes measured (**e**) before and (**f**) after CO_2RR. The error bars represent the standard deviation of three independent measurements.

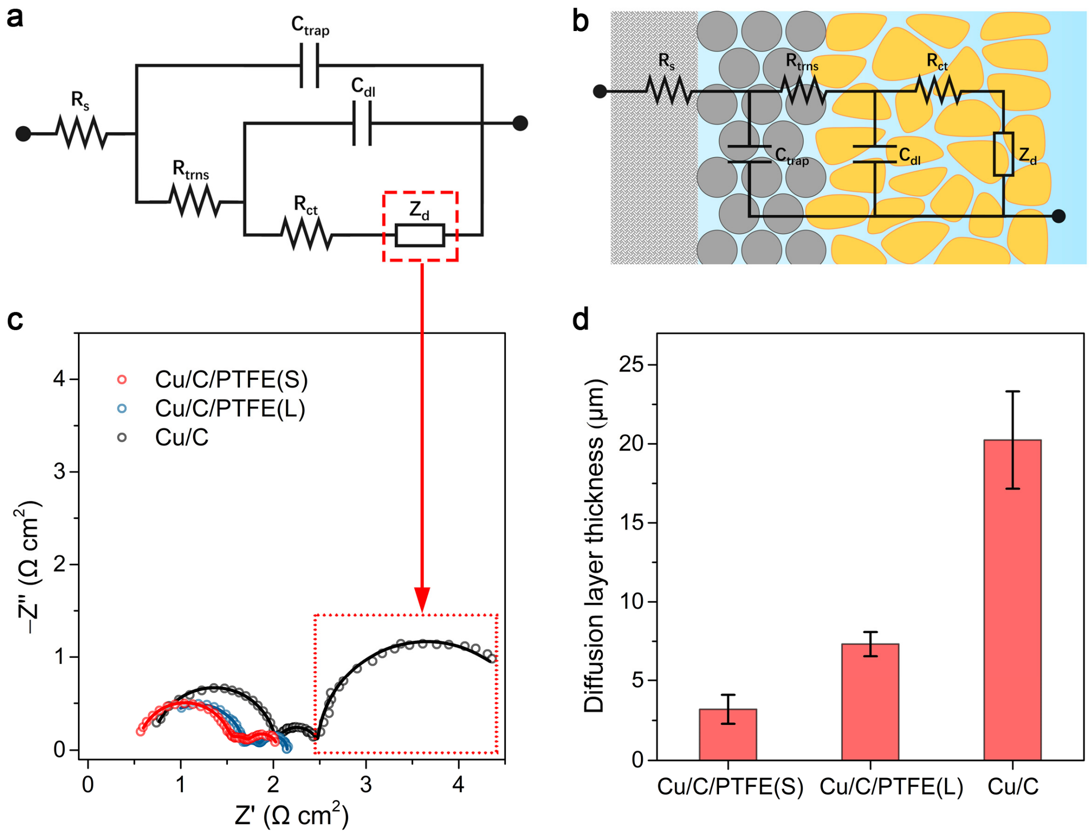

Supplementary Figure 18. Quantifying diffusion layer thickness using EIS. **a** A proposed circuit model and **b** its equivalent ladder circuit to describe the impedances in a porous carbon electrode[1], where R_s is internal resistance, R_{trns} is electron transport resistance in the porous carbon layer, C_{trap} is trap capacitance in the carbon layer, R_{ct} is charge transfer resistance at the electrode/electrolyte interface, C_{dl} is double layer capacitance at the electrode/electrolyte interface, Z_d is the impedance of the diffusion layer. **c** Nyquist plots obtained for the Cu/C, Cu/C/PTFE(S), and Cu/C/PTFE(L) electrodes under CO_2RR conditions at −1.0 V vs RHE in the GDE-cell. Symbols are experimental data and solid lines are fittings using the EIS Spectrum Analyser[2]. **d** Diffusion layer thickness δ in the three electrodes derived using equation[3]: $\delta = \sqrt{3R_d C_d D_0}$, where R_d and C_d are the equivalent resistance and capacitance of the diffusion layer representing the ability of conducting and storing electric charge of the layer, respectively, and D_0 is the diffusion coefficient of CO_2. R_d and C_d are determined from Z_d according to equation $Z_d \cong R_d - j\omega R_d^2 C_d$ at low frequencies[3], where j is the imaginary unit and ω is the angular frequency. The error bars represent the standard deviation of three independent measurements.

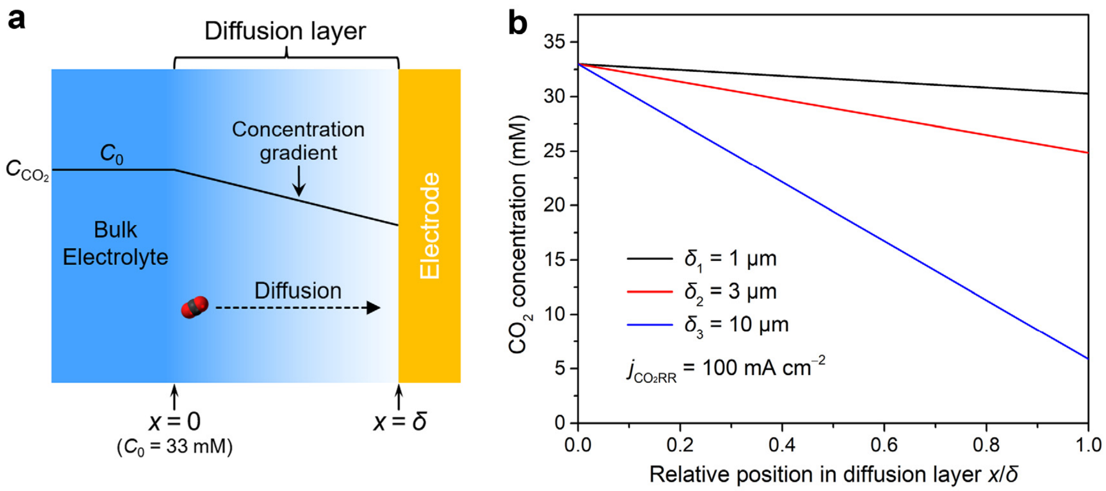

Supplementary Figure 19. a A geometric model explaining the diffusion layer, and **b** plot of the CO_2 concentration as a function of the relative position in the diffusion layer with the same CO_2RR current density (100 mA cm^{-2}) for different diffusion layer thicknesses, where $x = 0$ is the starting point of the diffusion layer and $x = \delta$ is the electrode surface. Other parameters (n, F, D_0, and C_0) are the same as those used in Supplementary Figure 1.

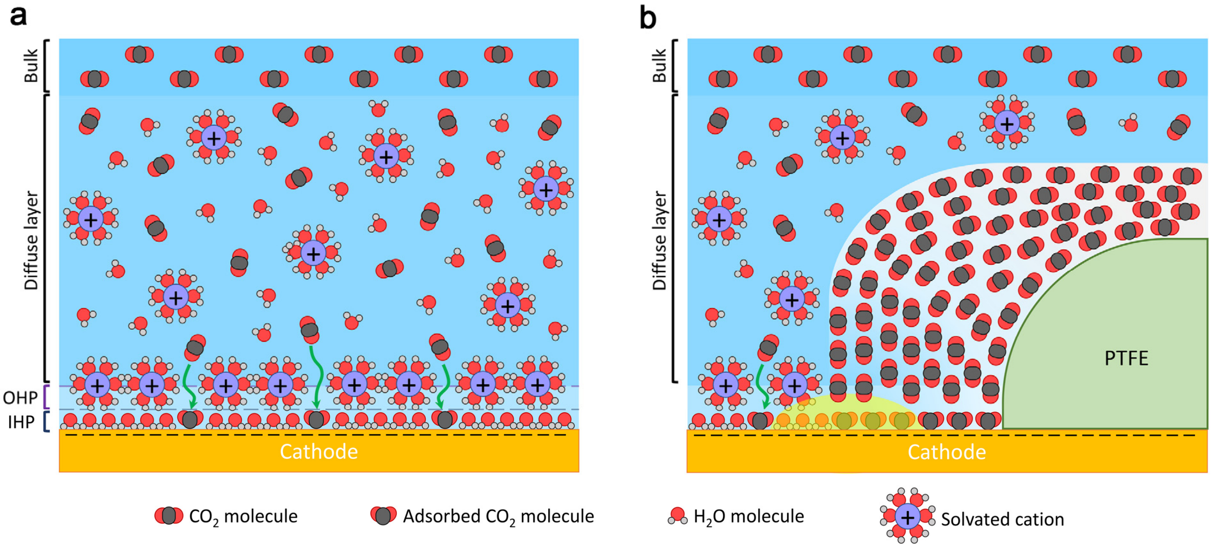

Supplementary Figure 20. Schematic illustration of the electrocatalytic interfaces for the CO_2RR: **a** conventional solid-liquid interface; **b** proposed solid-liquid-gas interface, where CO_2 gas trapped by PTFE near the catalyst enhances the adsorption of CO_2 and liquid electrolyte provides protons and ionic conductivity to promote CO_2RR at the three-phase boundary sites of the catalyst surface.

Supplementary References

1. Kwon, W., Kim, J. M. & Rhee, S. W. A new equivalent circuit model for porous carbon electrodes in charge transfer reaction of iodide/triiodide redox couples. *Electrochim. Acta* **68**, 110−113 (2012).
2. Bondarenko A. S. & Ragoisha G. A. In *Progress in Chemometrics Research*, Pomerantsev A. L., Ed. (Nova Science Publishers, New York, NY, 2005), p. 89−102.
3. Zhang, W. et al. Investigations on the interfacial capacitance and the diffusion boundary layer thickness of ion exchange membrane using electrochemical impedance spectroscopy. *J. Membr. Sci.* **502**, 37−47 (2016).
